# Single-cell profiling reveals three endothelial-to-hematopoietic transitions with divergent isoform expression landscapes

**DOI:** 10.1038/s44161-025-00740-z

**Published:** 2025-11-11

**Authors:** Wen Hao Neo, Muhammad Zaki Hidayatullah Fadlullah, Harshangda Bhatnagar, Cristiana Barone, Giulia Quattrini, Filipa Timóteo-Ferreira, Joana Carrelha, Gianluca Sala, Robert Sellers, John Weightman, Wolfgang Breitwieser, Natalia Moncaut, Roshana Thambyrajah, Sten Eirik W. Jacobsen, Mudassar Iqbal, Syed Murtuza Baker, Emanuele Azzoni, Michael Lie-A-Ling, Georges Lacaud

**Affiliations:** 1 Stem Cell Biology Group, Cancer Research UK Manchester Institute, The University of Manchester, Manchester, UK; 2 Department of Oncological Sciences, Huntsman Cancer Institute, University of Utah, Salt Lake City, UT USA; 3 Division of Informatics, Imaging and Data Sciences, Faculty of Biology, Medicine and Health, The University of Manchester, Manchester, UK; 4 School of Medicine and Surgery, University of Milano-Bicocca, Monza, Italy; 5 Haematopoietic Stem Cell Biology Laboratory, MRC Weatherall Institute of Molecular Medicine, University of Oxford, Oxford, UK; 6 Department of Immunology and Inflammation, Imperial College London, London, UK; 7 Computational Biology Support, Cancer Research UK Manchester Institute, The University of Manchester, Manchester, UK; 8 Molecular Biology Core Facility, Cancer Research UK Manchester Institute, The University of Manchester, Manchester, UK; 9 Genome Editing and Mouse Models, Cancer Research UK Manchester Institute, The University of Manchester, Manchester, UK; 10 Departamento de Biología Molecular, Facultad de Medicina, Universitat Cantabria, Santander, Spain; 11 Center for Hematology and Regenerative Medicine, Department of Medicine Huddinge & Department of Cell and Molecular Biology, Karolinska Institutet, Stockholm, Sweden; 12 Department of Hematology, Karolinska University Hospital, Stockholm, Sweden; 13 Fondazione IRCCS San Gerardo dei Tintori, Monza, Italy

**Keywords:** Haematopoietic stem cells, Cell lineage, Stem-cell differentiation, Haematopoietic stem cells, Differentiation

## Abstract

Hemogenic endothelium (HE) is recognized as the origin of all definitive blood cells, including hematopoietic stem cells (HSCs); however, the mechanisms governing the hematopoietic progenitor versus HSC fate choice within the HE remain unknown. Here we combine differentiation assays with full-length single-cell transcriptome data for extra-embryonic yolk sac (YS) and intra-embryonic aorta–gonad–mesonephros (AGM) region HE populations. We identified and localized three differentiation trajectories, each containing a distinct HE subset: erythromyeloid progenitor-primed HE in the YS plexus, lymphomyeloid progenitor-primed HE in large YS arteries and hematopoietic stem and progenitor cell-primed HE in the AGM. Chromatin modifiers and spliceosome components were enriched in AGM HE. This correlated with a higher isoform complexity of the AGM HE transcriptome. Distinct AGM HE-specific isoform expression patterns were observed for a broad range of genes, including stemness-associated factors like *Runx1*. Our data form a unique resource for studying cell fate decisions in different HE populations.

## Abstract (web summary)

Neo et al. map blood emergence from three hemogenic endothelial (HE) populations biased toward distinct blood fates. HE primed for stem progenitors shows elevated chromatin and RNA splicing gene expression and greater isoform diversity.

## Main

A pivotal step during mammalian ontogeny is the establishment of the hematopoietic system, which unfolds in three successive, partially overlapping waves^1,2^. The first two waves takes place in the yolk sac (YS). Wave 1 generates primitive erythrocytes and macrophages (E7.5)^3,4^. Wave 2 sequentially gives rise to erythromyeloid progenitors (EMPs; E8.25)^4,5^ and lymphomyeloid progenitors (LMPs; E9.5)^6,7^. The final wave, in the intra-embryonic aorta–gonad–mesonephros (AGM) region, produces hematopoietic stem and progenitor cells (HSPCs; wave-3, E10.5)^8,9^. In recent years, it has become evident that wave 2 cells not only play a role in wave 3 HSC generation but can also persist into adulthood^2,10,11^. The hematopoietic cells in wave 2 and wave 3, also known as the definitive waves, arise from a specific endothelium, called hemogenic endothelium (HE), through a process called endothelial-to-hematopoietic transition (EHT)^12–16^ orchestrated by the transcription factors RUNX1 (refs. ^13,17–19^) and GFI1 (refs. ^20,21^).

A critical question in hematopoietic development is why the HE in the extra-embryonic space is skewed toward EMP and LMP generation, whereas intra-embryonic HE, primarily localized in the dorsal aorta, can efficiently give rise to HSCs. The spatiotemporal difference in emergence suggests that HE cells from distinct waves are intrinsically different, leading to divergent molecular dependencies. Indeed, NOTCH signaling is essential for HSC development but not for EMP generation^22,23^. Conversely, *Ezh2* is essential for the generation of functional EMPs, whereas it is dispensable for AGM HSC development^24,25^.

Single-cell RNA sequencing (scRNA-seq) is ideally suited to identify intrinsic differences between rare cell populations. We previously characterized a granular full-length (Smart-seq2) single-cell transcriptomic profile of the AGM EHT trajectory, defining a HE continuum (HE^AGM^) encompassing HE cells at various stages of commitment^26^. Here, we present the acquisition and analysis of a complementary full-length transcriptome of extra-embryonic EHT populations. We identified two distinct extra-embryonic HE populations, both residing within the *Runx1*^pos^KIT^pos^ endothelial population. The first, HE^YSP^, is contained within CD24^neg^*Vwf*^neg^ LYVE1^pos^ endothelial cells, is dominant before E9.5, has EMP potential, and is localized throughout the YS endothelial plexus. The second, HE^YSA^, is contained within CD24^pos^*Vwf*^pos^ LYVE^neg^ endothelial cells, is dominant after E9.5, has LMP potential, and is exclusively found in large extra-embryonic arteries.

Our data reveal both striking similarities and differences between extra- and intra-embryonic HE populations. While all HE populations share a common signature marked by the expression of the transcription factors *Gfi1* and *Mycn*, there are pronounced differences with regard to the expression of chromatin modifiers and genes involved in RNA processing. This correlates with increased isoform complexity in the HE^AGM^ transcriptome. Distinct HE^AGM^-specific isoform expression patterns are observed across a broad range of genes, suggestive of a stochastic transcriptional environment guiding the unique HSPC cell fate choices made within the AGM. Notably, multiple stemness-associated factors, such as *Runx1*, display differential isoform expression profiles when compared to the YS HE populations.

The dataset presented here forms a comprehensive full-length scRNA-seq atlas of three distinct definitive hematopoietic EHT trajectories giving rise, respectively to EMPs, LMPs and HSPCs, which can be accessed and queried at https://shiny.cruk.manchester.ac.uk/AGM_YS_dataset_final/.

## Results

### Extra-embryonic HE potential resides within the KIT^pos^ population

**Fig. 1:**
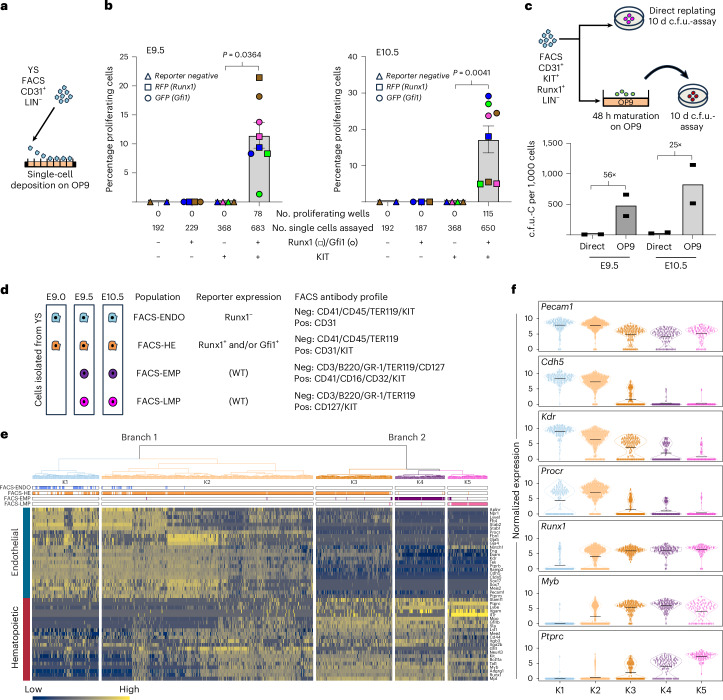
Single-cell profiling of the extra-embryonic KIT^pos^ endothelial fraction to characterize extra-embryonic EHT. **a**–**c**, Extra-embryonic HE potential resides within the KIT^pos^ population. Schematic of single-cell hematopoietic assays on the CD31^pos^Lineage^neg^ (CD41^neg^CD45^neg^TER119^neg^) extra-embryonic populations (**a**). Sorted KIT^pos^ and KIT^neg^ extra-embryonic single cells were co-cultured on OP9 feeder cells for 7 days. Hematopoietic activity was only observed in KIT^pos^RUNX1^pos^ and KIT^pos^RUNX1^pos^GFI1^pos^ cells (**b**). No hematopoietic activity was observed in either the KIT^pos^RUNX1^neg^ or the KIT^neg^RUNX1^pos^ cells. Squares represent *Runx1* positive cells isolated from *Runx1*:RFP reporters, circles represent *Runx1/Gfi1* double positive cells isolated from *Runx1*:RFP/*GFi1*:GFP double reporters, triangle indicate *Runx1 / Gfi1* negative cells. Different biological experiments (for each reporter used) are indicated by color (brown, blue, green, magenta). KIT^pos^ cells were obtained from *n* = 4 biological experiments, KIT^neg^ cells were obtained from *n* = 2 biological experiments. Bars represent the average percentage of proliferating cells ± s.e.m. Statistical test was a two-tailed paired *t*-test. Error bars are not displayed for reporter KIT^neg^ samples and these samples were not tested for statistical significance. Hematopoietic colony-forming unit (c.f.u.) assay on KIT^pos^Runx1^pos^CD31^pos^Lin^neg^ (CD41^neg^CD45^neg^TER119^neg^) extra-embryonic cells (**c**). Cells were either directly replated or co-cultured with OP9 feeder cells for 48 h before replating. Hematopoietic colonies were quantified after 10 days. *n* = 2 biological experiments. Bars represent the average number of c.f.u. per 1000 cells seeded. Numbers above the bars represent the fold increase in hematopoietic output. **d**–**f**, Single-cell profiling of extra-embryonic EHT. Schematic of the cell populations FACS sorted from dissected E9, E9.5 and E10.5 YS and processed for full-length single-cell Smart-seq2 RNA sequencing (**d**). Endo, endothelium. Tree dendrogram generated by hierarchical clustering of the sorted populations in **c** (**e**). Two main branches are identified (K1–K2 and K3–K5). Below the dendrogram, the contribution of the different FACS-sorted populations to each cluster is shown. Bottom: heatmap depicting the expression of endothelial (top) and hematopoietic (bottom) genes across clusters K1–K5. Violin plots depicting the expression of selected endothelial (*Pecam1*, *Cdh5*, *Kdr* and *Procr*) and hematopoietic genes (*Runx1*, *Myb* and *Ptprc*) across clusters K1–K5 (**f**). Black bars represent the mean expression level. Source Data

**Extended Data Fig. 1:**
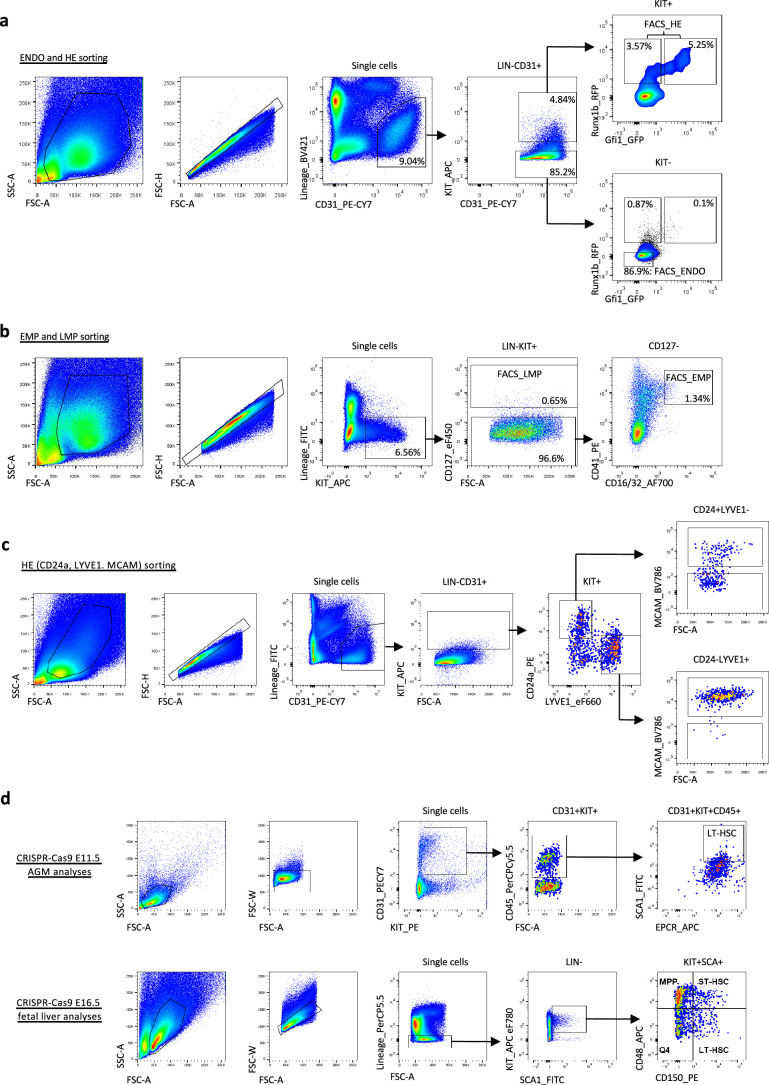
FACS sorting profiles used in this study. **a**, Gating strategy for endothelial and hemogenic endothelial sorts from yolk sac (YS). To determine which populations in the YS contain hemogenic potential, the following populations were sorted and analyzed for hematopoietic activity *in vitro:* CD41^neg^CD45^neg^TER119^neg^CD31^pos^KIT^neg^Runx1b:RFP^pos^ CD41^neg^CD45^neg^TER119^neg^CD31^pos^KIT^neg^Runx1b:RFP^pos^Gfi1:GFP^pos^ CD41^neg^CD45^neg^TER119^neg^CD31^pos^KIT^pos^Runx1b:RFP^pos^ CD41^neg^CD45^neg^TER119^neg^CD31^pos^KIT^pos^Runx1b:RFP^pos^Gfi1:GFP^pos^ Non-hemogenic endothelial cells (FACS-ENDO) for scRNA-seq were sorted as: CD41^neg^CD45^neg^TER119^neg^CD31^pos^KIT^neg^Runx1b:RFP^neg^ Hemogenic endothelium enriched (FACS-HE) populations for scRNA-seq were sorted as: CD41^neg^CD45^neg^TER119^neg^CD31^pos^KIT^pos^**R**unx1b:RFP^pos^**G**fi1:GFP^pos^ CD41^neg^CD45^neg^TER119^neg^CD31^pos^KIT^pos^**G**fi1:GFP^pos^ CD41^neg^CD45^neg^TER119^neg^CD31^pos^KIT^pos^**R**unx1b:RFP^pos^Gfi1:GFP^neg^ **b**, Gating strategy for erythromyeloid progenitors (EMP) and lymphomyeloid progenitors (LMP) sorts from YS. FACS-EMP: CD3^neg^B220^neg^GR1^neg^TER119^neg^KIT^pos^CD127^neg^CD41^pos^CD16/32^pos^. Bertrand, J.Y. et al. Blood 106, 3004-3011 (2005). Frame, J.M., Fegan, K.H., Conway, S.J., McGrath, K.E. & Palis, J. Stem Cells 34, 431-444, 2016. FACS-LMP: CD3^neg^B220^neg^Gr1^neg^TER119^neg^KIT^pos^CD127^pos^ Boiers, C. et al. Cell Stem Cell 13, 535-548, 2013. Yoshimoto, M. et al. Proc Natl Acad Sci U S A 108, 1468-1473, 2011. **c**, Gating strategy to enrich for HE with LMP potential and HE with EMP potential from YS. FACS-HE^LMP:^CD41^neg^CD45^neg^TER119^neg^ CD31^pos^KIT^pos^CD24a^pos^LYVE1^neg^MCAM^neg^ FACS-HE^EMP^:CD41^neg^CD45^neg^TER119^neg^ CD31^pos^KIT^pos^CD24a^neg^LYVE1^pos^MCAM^neg^ **d**, Gating strategy to analyze phenotypic long-term HSC (LT-HSC) and emerging hematopoietic cells (CD31^pos^KIT^pos^CD45^pos^) in E11.5 AGM regions and long-term HSC (LT-HSC) in E16.6 fetal livers. LT-HSC (AGM): CD31^pos^SCA^pos^KIT^pos^CD45^pos^EPCR^pos^. LT-HSC (fetal liver): B220^neg^CD3^neg^GR1^neg^TER119^neg^CD48^neg^SCA^pos^KIT^pos^CD150^pos^.

**Extended Data Fig. 2:**
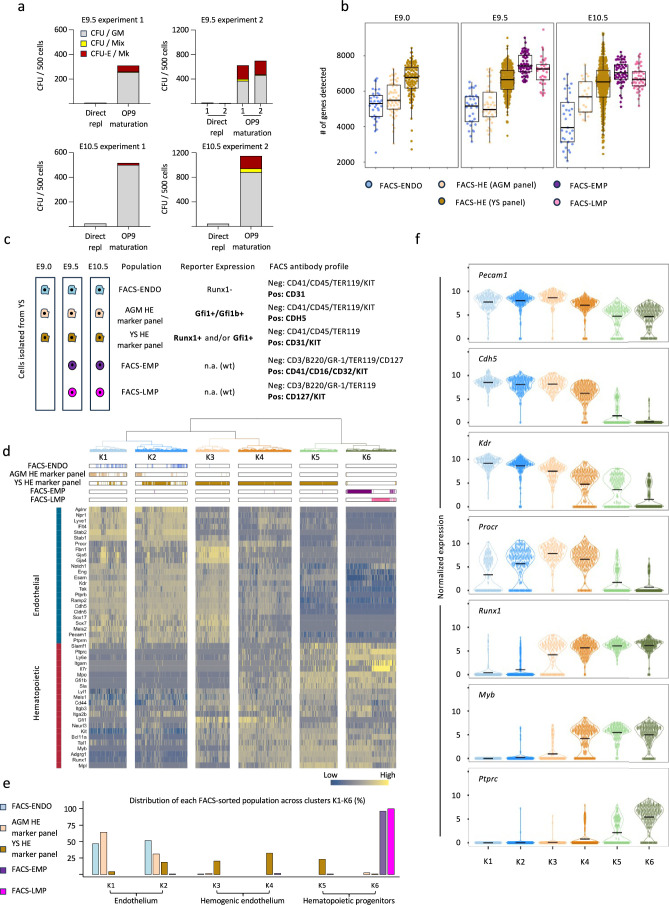
Identification of HE activity in KIT^pos^Runx1^pos^CD31^pos^LIN^neg^ extra-embryonic cells for single-cell profiling of extra-embryonic EHT. **a**, Hematopoietic colony forming unit (CFU) assay on KIT^pos^Runx1^pos^CD31^pos^LIN^neg^ extra-embryonic cells FACS-isolated from Runx1^RFP^ reporter mice. Cells were either directly replated, or co-cultured with OP9 feeder cells for 48 h before replating. Hematopoietic colonies were quantified after 10 days. Individual CFU assays are shown. N = 2 biological experiments. E/Mk = Erythrocyte / Megakaryocyte, Mix = Granulocyte / Erythrocyte / Macrophage / Megakaryocyte, G/M = Granulocyte / Macrophage. **b**, Numbers of genes detected and the number of cells that passed QC within each FACS-sorted population from dissected E9.0, E9.5 and E10.5 YS (full-length scRNA-seq, Smart-seq2). Embedded boxplots indicate the median (horizontal line), the upper and lower hinges represent the 75^th^ and 25^th^ percentile and whiskers extend to 1.5x the inter-quartile range. **c**, Schematic of the cell populations FACS-sorted from dissected E9, E9.5 and E10.5 YS and processed for full-length single-cell Smart-seq2 RNA sequencing. Endo: endothelium, AGM HE marker panel (CD41^neg^CD45^neg^TER119^neg^CDH5^pos^KIT^neg^Gfi1/Gfi1b^pos^), YS HE marker panel (CD41^neg^ CD45^neg^ TER119^neg^CD31^pos^KIT^pos^Runx1/Gfi1^pos^), EMP: erythro-myeloid progenitor, LMP: lympho-myeloid progenitor. **d**, Tree dendrogram generated by hierarchical clustering of the sorted populations in (c). Below the dendrogram, the contribution of the different FACS-sorted populations to each cluster is shown. All cells sorted from the YS using the AGM HE marker panel cluster together with YS FACS-ENDO cells. Bottom: heatmap depicting the expression of endothelial (top) and hematopoietic (bottom) genes across clusters K1-K6 **e**, Bar graph depicting the distribution (as percentage) of each FACS-sorted population listed in (c) across clusters K1-K6. **f**, Violin plots depicting the expression of selected endothelial (*Pecam1*, *Cdh5*, *Kdr*, *Procr*) and hematopoietic genes (*Runx1*, *Myb*, *Ptprc*) across clusters K1-K6. Black bars represent the mean expression level. Source Data

Although HE activity was previously reported to reside within KIT^neg^ cells in the AGM^12,27^, it is associated with KIT^pos^ cells during in vitro mouse embryonic stem cell differentiation^16^, recapitulating YS hematopoiesis^16^. To determine whether extra-embryonic HE is mainly found within the KIT^neg/low^ or KIT^pos^ endothelium (defined as CD31^pos^ and hematopoietic lineage/LIN^neg^:CD41^neg^CD45^neg^TER119^neg^), we examined the hematopoietic potential of E9.5 and E10.5 YS endothelial cells from *Runx1b*^RFP^/*Gfi1*^GFP^ reporter mice^26,28,29^. *Runx1* and *Gfi1* expressions are robust indicators of HE identity^20,21,26,28^. YS KIT^neg/low^ or KIT^pos^ FACS-sorted single endothelial cells were co-cultured on OP9 cells for 7 days to support EHT and hematopoietic expansion (Fig. 1a and Extended Data Fig. 1a). Hematopoietic potential was only detected in wells seeded with endothelial cells expressing KIT and RUNX1 (Figs. 1b; E9.5 and E10.5). No hematopoietic cells were generated from either the KIT^pos^*Runx1*^neg^ or KIT^neg^ endothelial populations. Robust colony formation of the CD41^neg^CD45^neg^TER119^neg^CD31^pos^KIT^pos^Runx1b:RFP^pos^ cells was only observed after maturation/EHT on OP9 cells (Fig. 1c and Extended Data Figs. 1a and 2a) indicating that this population contains true HE cells and not already committed hematopoietic cells. Together, these data establish that at E9.5 and E10.5 YS extra-embryonic HE predominantly resides within the KIT^pos^*Runx1*^pos^CD31^pos^LIN^neg^ population.

### scRNA-seq profiling of the extra-embryonic EHT trajectory

To construct a comprehensive full-length Smart-seq2 scRNA-seq dataset capturing the extra-embryonic EHT process, akin to our previous AGM HE study^26^, we isolated individual cells of extra-embryonic populations across E9.0, E9.5 and E10.5. These included cells from HE-enriched (FACS-HE) populations from single and double *Runx1* and *Gfi1* reporter mice, non-HE endothelial cells (FACS-ENDO), and committed EMP (FACS_EMP) and LMP (FACS-LMP) hematopoietic progenitors (Fig. 1d and Extended Data Fig. 1a,b). Overall, 960 sequenced cells (100 FACS-ENDO, 660 FACS-HE, 118 EMP and 82 LMP) passed quality control with a median of 6,553 genes detected per individual cell (Extended Data Fig. 2b and Methods).

Unsupervised hierarchical clustering separated the cells into five clusters (K1–K5), with two main dendrogram branches (Fig. 1e). The first branch (K1–K2) exhibits a strong endothelial identity, with K1 containing the majority of FACS-ENDO (Fig. 1e-f). The second branch (K3–K5) has a pronounced hematopoietic identity, with the FACS-EMP and the FACS-LMP cells localizing within K4 and K5, respectively (Fig. 1e,f). K3 also displays a strong hematopoietic profile, including expression of *Ptprc* (*CD45*) and *Myb*, and markedly reduced expression of endothelial genes (*Cdh5*, *Kdr/Flk1*, *Pecam1* and *Procr*) compared to K1–K2 (Fig. 1e,f). These data indicate that HE cells reside in K2, and that K3 consists of committed early hematopoietic progenitors.

Finally, we reclustered the above YS populations with the addition of 115 YS cells, which were sorted using the established AGM HE phenotype: KIT^neg^ CD41^neg^CD45^neg^CDH5^pos^*Gfi1/*^*Gfi1b*pos^ (refs. ^21,26^). More than 95% of these cells clustered together with FACS-ENDO cells, further confirming that within the YS, HE resides within the KIT^pos^ population (Extended Data Fig. 2c–f). Altogether, these analyses suggest that we captured the full extra-embryonic YS EHT process.

### Integration of YS and AGM scRNA-seq data reveals three distinct EHT trajectories

**Fig. 2:**
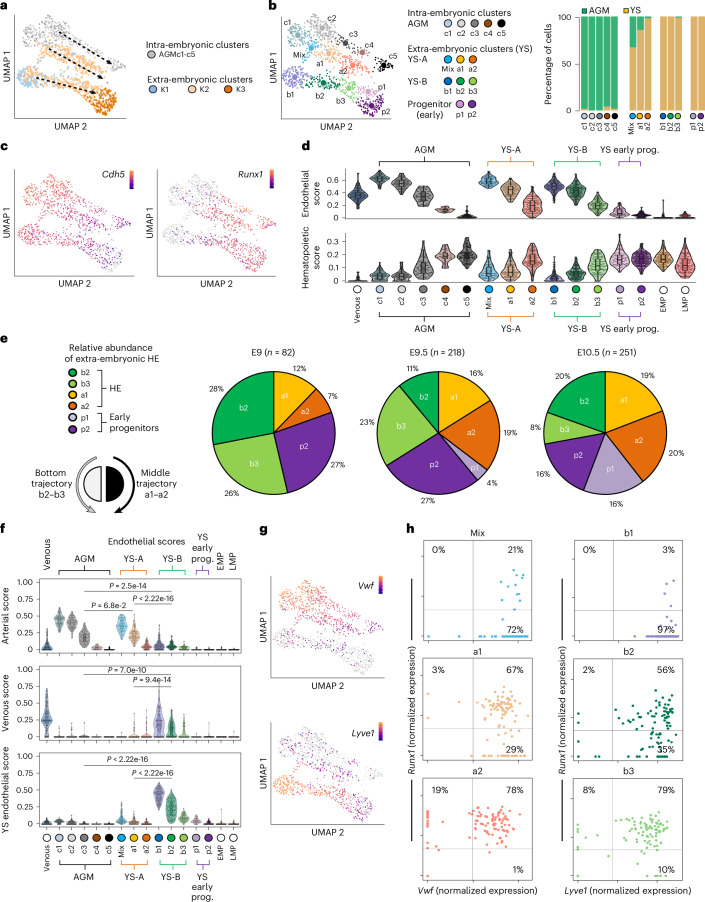
Two extra-embryonic EHT trajectories with distinct endothelial signatures. Semi-supervised clustering of intra-embryonic (AGM-derived) and extra-embryonic EHT scRNA-seq datasets. **a**, UMAP of the integrated data overlayed with the K1–K3 extra-embryonic YS clusters defined in Fig. 1d. Arrows indicate the presence of three EHT trajectories (one AGM trajectory and two YS trajectories) **b**, UMAP of the integrated data depicting the 13 clusters spread across intra-embryonic (AGM-derived) and extra-embryonic cells (YS-derived) cells (left). Clusters AGMc_3 and AGMc_4 form the intra-embryonic/AGM HE continuum (HE^AGM^). There are two putative HE continua in the extra-embryonic space: YSc_a1, YSc_a2 and YSc_b2, YSc_b3. Graph depicting the contribution of intra-embryonic derived cells (AGM) and extra-embryonic derived cells (YS) to each cluster (right). **c**, UMAPs depicting the expression of the endothelial gene *Cdh5* and the hematopoietic gene *Runx1*. **d**, Violin plot depicting endothelial (top) and hematopoietic (bottom) signature scores across all 13 clusters defined in **b**. The signature scores were calculated using the genes depicted in Fig. 1d. Embedded boxplots indicate the median (horizontal line), the upper and lower hinges represent the 75th and 25th percentile and whiskers extend to 1.5 × interquartile range. **e**, Relative abundance of extra-embryonic clusters YSc-b2 YSc-b3, YSc-a1 YSc-a2 (putative HE) and YSc-p1, YSc p2 (early hematopoietic progenitors). Numbers depict the percentage of the total number FACS-HE cells across all analyzed clusters at each embryonic stage. **f**, Violin plots depicting arterial, venous and YS endothelial scores across all clusters defined in **b**. For reference, AGM-derived venous endothelial cells (left column) and extra-embryonic-derived EMP and LMP populations (right columns) are also included. Embedded boxplots indicate the median (horizontal line), the upper and lower hinges represent the 75th and 25th percentile and whiskers extend to 1.5 × interquartile range. Two-sided Wilcoxon rank-sum tests were used (with *P* values adjusted via the Benjamini–Hochberg procedure to control the FDR) to compare the early HE clusters (c3, a1 and b2). **g**, UMAPs depicting the expression of the *Vwf* (marking the AGM and YS-A clusters) and *Lyve1* (marking YS-B clusters). **h**, Correlation between transcript expression of *Runx1* and *Vwf* in clusters mix, a1 and a2 (YS-A trajectory) (top). Correlation between *Runx1* and *Lyve1* transcript expression in clusters b1, b2 and b3 (YS-B trajectory) (bottom). Source Data

**Extended Data Fig. 3:**
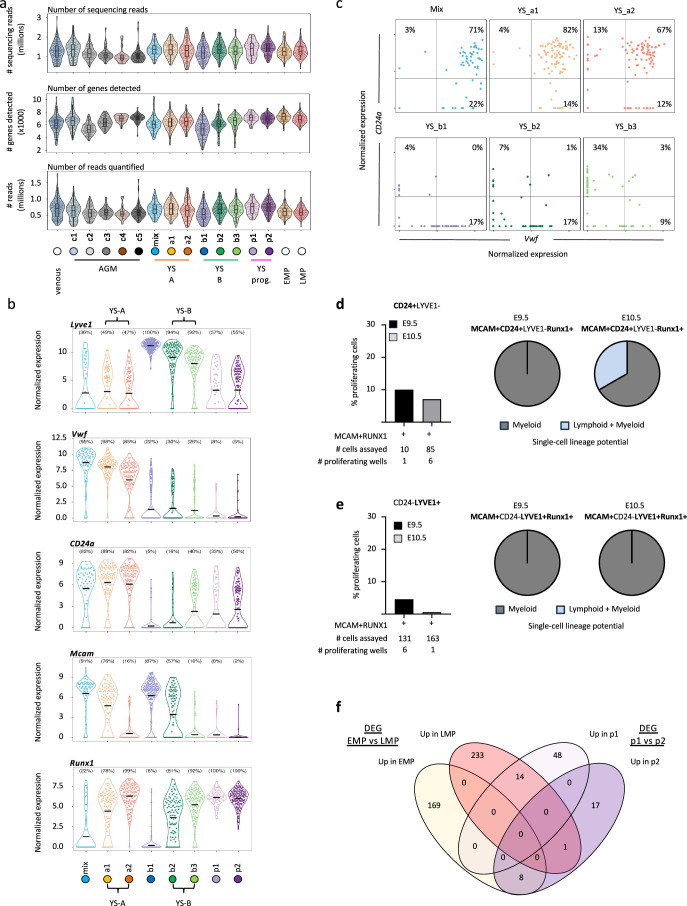
Two distinct extra-embryonic EHT trajectories. **a**, Violin plots depicting the # of sequencing reads, # of genes detected, # of reads quantified for all scRNA-seq clusters/populations analyzed in this study. Embedded boxplots indicate the median (horizontal line), the upper and lower hinges represent the 75^th^ and 25^th^ percentile and whiskers extend to 1.5x the inter-quartile range. The AGM-derived part of the dataset is described in depth in Fadlullah, M.Z.H. et al. *Blood* 139, 343-356, 2022. **b**, Violin plots depicting the expression of candidate genes for immunofluorescence- based analyses of the YS-middle and YS-bottom clusters. **c**, Top: Correlation between transcript expression of *CD24a* and *Vwf* in YS-middle trajectory clusters (mix-m1-m2) and YS-bottom trajectory clusters (b1-b2-b3). Both *Vwf* and *CD24a* are selective for clusters mix, m1 and m2. **d**, Left: Single-cell hematopoietic assays of early YS-middle cells (likely cluster m1) (KIT^pos^CD31^pos^LIN^neg^LYVE1^neg^CD24^pos^MCAM^pos^Runx1:RFP^pos^) cultured on OP9 feeder cells for 14 days. The percentage of wells containing proliferating hematopoietic cells is shown. Right: Lineage distribution of the hematopoietic cells shown in the left panel, as determined by flow cytometry for myeloid (GR1, MAC1/CD11b*)*, erythroid (TER119) and lymphoid (CD19) markers. No erythropoiesis was observed. **e**, Left: Single-cell hematopoietic assays of early YS-bottom cells (likely cluster b2) (KIT^pos^CD31^pos^LIN^neg^LYVE1^pos^CD24^neg^MCAM^pos^Runx1:RFP^pos^) cultured on OP9 feeder cells for 14 days. The percentage of wells containing proliferating hematopoietic cells is shown. Right: Lineage distribution of the hematopoietic cells shown in the left panel, as determined by flow cytometry for myeloid (GR1, MAC1/CD11b*)*, erythroid (TER119) and lymphoid (CD19) markers. No erythropoiesis was observed. **f**, EMP-fate (8 genes) and LMP-fate (14 genes) signatures were extracted by intersecting pairwise differential gene expression results (EMP vs LMP and P1 vs P2). Fate signatures are listed in Supplementary Table 1. Source Data

To compare extra-embryonic with intra-embryonic EHT, we conducted a joint analysis with our previously published AGM EHT dataset^26^ (Extended Data Fig. 3a). We utilized a semi-supervised clustering approach and focused on populations that retain some endothelial characteristics: extra-embryonic clusters K1–K3 and AGM CDH5^pos^ clusters (Fig. 2a,b). The data integration revealed three parallel sets of EHT clusters (Fig. 2b–d) with minimal overlap between extra-embryonic and AGM-derived cells (Fig. 2a,b). We designated the three trajectories as trajectory A and B for the YS-derived cells and trajectory C for the AGM-derived cells. Overall, the integration resulted in 13 clusters (Fig. 2b), which were named based on their known identity/trajectory within the Uniform Manifold Approximation and Projection (UMAP); AGM clusters^26^ (c1_arterial endothelium, c2_pre-HE, c3_HE, c4_EHT, c5_intra-aortic-hematopoietic-clusters), YS-A trajectory clusters (a1 and a2), YS-B trajectory clusters (b1, b2 and b3), YS-progenitor clusters (p1 and p2). The only cluster that demonstrated an appreciable overlap between YS and AGM-derived cells was called Mix (Fig. 2b). Cells from YS endothelial (K1) and hematopoietic (K3) clusters, respectively, contributed to b1 and p1/p2 populations. Most cells from the YS HE population (K2) contributed to two distinct pairs of clusters (a1–a2 and b2–b3), situated parallel to AGM c3_HE and c4_EHT. Another scRNA-seq profiling study annotated cells similar to c3_HE as pre-HE, and c4_EHT as HE^26,30^. To reconcile semantic differences in HE definitions across studies, we considered both c3 and c4 as a single HE entity or continuum (HE^AGM^). Using HE^AGM^ and the coexpression of hematopoietic and endothelial genes (Fig. 2c,d), we inferred that extra-embryonic clusters a1, a2, b2 and b3, likely possess HE properties. Overall, the integration of AGM and extra-embryonic EHT datasets suggests the existence of three distinct EHT trajectories.

### Differential spatiotemporal emergence of extra-embryonic EHT trajectories

To unravel the characteristics of the extra-embryonic EHT clusters, we first examined the relative prevalence of each cluster present in the FACS-HE population from E9.0 to E10.5 (Figs. 1d–f and 2e). Cells in clusters b2–b3 were more prevalent at earlier (E9.0) developmental stages than cells in a1–a2, suggesting that the YS-B EHT trajectory is established before the YS-A trajectory. The appearance of the two extra-embryonic progenitor clusters followed a similar sequential pattern, with p2 preceding p1.

As endothelial gene expression plays a pivotal role in defining HE identity, we evaluated whether the different trajectories could be segregated based on endothelial profiles (arterial, venous and YS) (Fig. 2f and Supplementary Table 1). The AGM EHT trajectory exhibited a robust arterial endothelial identity, similar to extra-embryonic clusters mix, a1 and a2, whereas b1, b2 and b3 displayed venous and especially YS endothelial profiles (Fig. 2f). Mix and b1 likely represent non-HE endothelial cells, as they display the strongest arterial and YS endothelial profiles within their respective trajectories while also lacking *Runx1* expression in most of the cells that make up the cluster (Fig. 2c,f and Extended Data Fig. 3b).

**Fig. 3:**
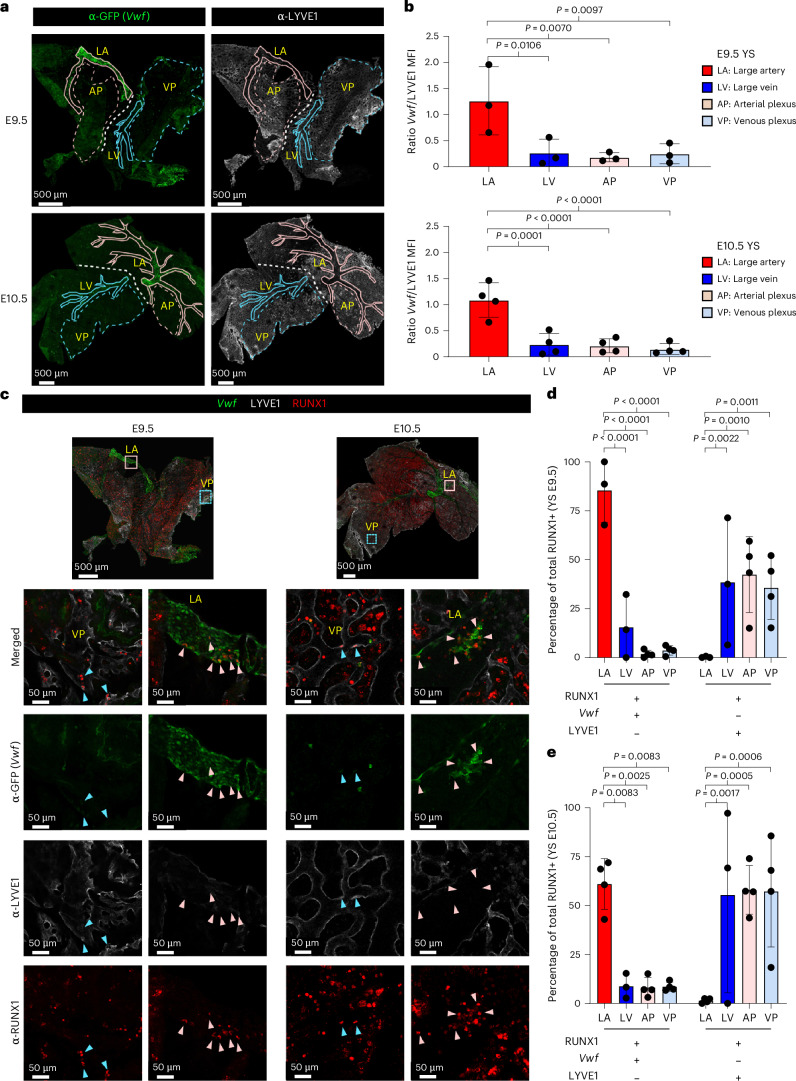
Spatial separation between transcriptomically different EHT trajectories in the yolk sac. **a**, Confocal whole-mount immunofluorescence (WM IF) analysis of E9.5 (top) and E10.5 (bottom) *Vwf*^eGFP^ YS. Images show maximum intensity three-dimensional (3D) projections. Representative areas where fluorescence has been quantified are delimited by lines. Pink solid line, large artery (LA); turquoise solid line, large vein (LV); pink dashed line, arterial plexus (AP); turquoise dashed line, vein plexus (VP). Scale bars, 500 µm. **b**, The ratio of *Vwf*-associated MFI to LYVE1-associated MFI is plotted on the *y* axis, reflecting the relative fluorescence intensities within selected areas in *Vwf*^eGFP^ YS as displayed in Fig. 3a. *n* = 3 E9.5 and *n* = 4 E10.5 YS were analyzed (6–10 areas per YS). Error bars represent mean ± s.d. Statistical test used was a one-way analysis of variance (ANOVA) (Fisher’s least significant difference). **c**, WM IF analysis of E9.5 (left) and E10.5 (right) *Vwf*^eGFP^ YS. Whole YS images show maximum intensity 3D projections. The boxed area in the merged image is magnified in the lower panel and shows a single 2.5-mm-thick optical slice. Turquoise arrowheads indicate RUNX1^pos^*Vwf*^neg^LYVE1^pos^ putative HE cells; pink arrowheads indicate RUNX1^pos^*Vwf*^pos^LYVE1^neg^ putative HE cells. Scale bars, 500 µm (3D), 50 µm (slice). **d**,**e**, Quantification of the percentage of RUNX1^pos^*Vwf*^pos^LYVE1^neg^ and RUNX1^pos^*Vwf*^neg^LYVE1^pos^ cells on the total of RUNX1 positive cells in LA, LV, AP and VP of E9.5 (**d**) and E10.5 (**e**) *Vwf*^eGFP^ YS (displayed in Fig. 3c). Each dot represents measurements from an individual YS. E9.5 LA and LV *n* = 3, AP and VP *n* = 4. E10.5 LV *n* = 3, LA, AP and VP *n* = 4 5–17 areas per YS were analyzed. Error bars represent mean ± s.d. Statistical test used was a two-way ANOVA (Fisher’s least significant difference). Source Data

Finally, we screened for specific markers allowing us to determine the spatial localization, within the YS, of cells representing these different EHT trajectories. Differential gene expression analysis identified the endothelial genes *Vwf* (Von Willebrand factor) and *CD24a* (a glycosylphosphatidylinositol (GPI)-anchored cell surface protein) as good markers for the YS-A trajectory. *Lyve1* (lymphatic vessel endothelial hyaluronan receptor 1) was associated with the YS-B trajectory (Fig. 2g,h and Extended Data Fig. 3c). Whole-mount staining of E9.5 and E10.5 YS, obtained from a *Vwf*^eGFP^ reporter^31^ mouse model, for RUNX1, LYVE1 and eGFP revealed their distinct spatial expression patterns within the extra-embryonic vasculature. While high LYVE1 expression was evident throughout the YS plexus and in large veins, eGFP (*Vwf* expression) staining was confined to large arterial vessels (Fig. 3a,b). Additionally, putative HE cells expressing both RUNX1 and *Vwf* were primarily observed in large arteries and infrequently in large veins. In the plexus RUNX1^pos^*Vwf*^pos^ cells were absent at E9.5 and infrequent at E10.5. Putative HE cells expressing RUNX1 and LYVE1 were distributed throughout the plexus (Fig. 3c–e). Altogether, these results suggest the presence of two separate extra-embryonic HE populations. The first (b2 and b3) is LYVE1 positive, dominant until E9.5 and can be found throughout the YS endothelial plexus. The second population (a1 and a2) expresses CD24a and *Vwf*, is prevalent after E9.5 and is found in large extra-embryonic arteries.

### The two extra-embryonic HE populations have distinct hematopoietic potentials

**Fig. 4:**
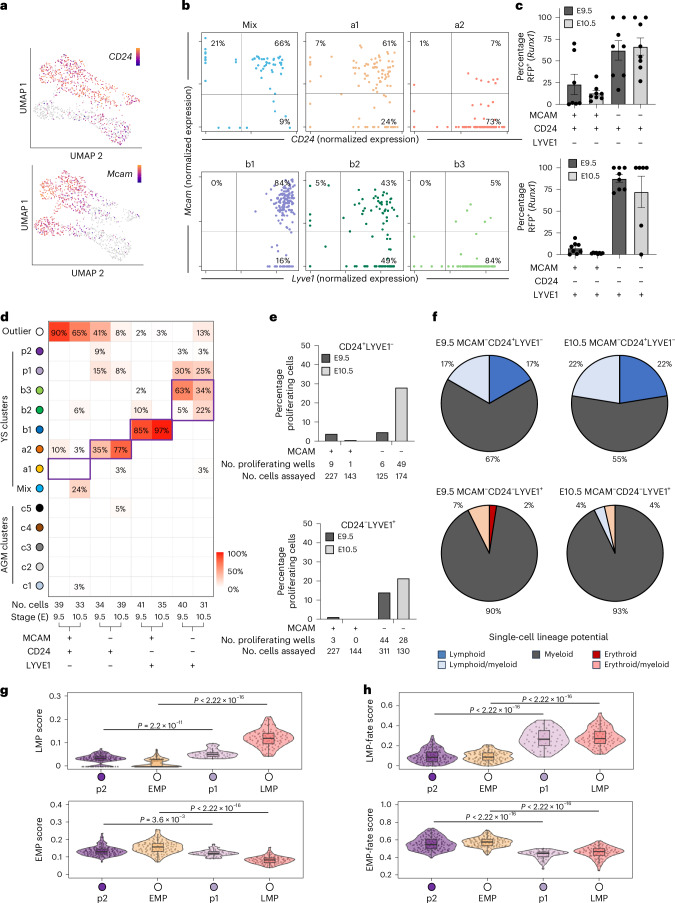
CD24^pos^ YS HE has lymphoid-myeloid potential and LYVE1^pos^ YS HE has erythroid-myeloid potential. **a**, UMAPs depicting the expression of *Cd24a* (marking the AGM and YS-A clusters) and *Mcam* (marking cells toward the endothelial end of all three trajectories). **b**, Correlation between *Cd24a* and *Mcam*, transcript expression in clusters mix, a1 and a2 (YS-A trajectory) (top). Correlation between *Lyve1* and *Mcam*, transcript expression in clusters b1, b2 and b3 (YS-B trajectory) (bottom). **c**, Flow cytometry on extra-embryonic CD45^neg^CD41^neg^TER119^neg^ (Lineage negative) CD31^pos^KIT^pos^ cells from *Runx1*:RFP reporter embryos. MCAM, LYVE1 and CD24 antibodies were used to analyze the proportion of *Runx1* (RFP) expressing cells in different subpopulations. Each dot represents cells from a single YS. E9.5 MCAM^pos^CD24^pos^LYVE1^neg^ *n* = 7, E10.5 MCAM^neg^CD24^neg^LYVE1^pos^ *n* = 6, all other samples *n* = 8. Bars represent the average ± s.e.m. **d**, Heatmap displaying the distribution (as percentage) of different CD45^neg^CD41^neg^TER119^neg^CD31^pos^KIT^pos^ FACS-sorted, scRNA-sequenced cell populations across the in silico EHT clusters defined in Fig. 2b. Based on *k*-nearest-neighbor classifier approach. MCAM, LYVE1, CD24 sorting profiles are depicted on the *x* axis. Purple boxes indicate the expected/predicted cluster (*y* axis) for the sorted population (*x* axis) based on the data presented in Fig. 4b. **e**, Single-cell hematopoietic assays of YS-A HE (KIT^pos^CD31^pos^LIN^neg^LYVE1^neg^CD24^pos^MCAM^neg^) and YS-B HE (KIT^pos^CD31^pos^LIN^neg^LYVE1^pos^CD24^neg^MCAM^neg^) cultured on OP9 feeder cells for 14 days. The percentage of wells with proliferating hematopoietic cells is shown. **f**, Lineage distribution of the hematopoietic cells shown in **e** as determined by flow cytometry for myeloid (GR1 and MAC1/CD11b), erythroid (TER119) and lymphoid (CD19) markers. **g**, Violin plots depicting LMP (top) and EMP (bottom) scores across early progenitor clusters p1 and p2. EMP and LM signatures have been previously published and are listed in Supplementary Table 1. Embedded boxplots indicate the median (horizontal line), the upper and lower hinges represent the 75th and 25th percentile and whiskers extend to 1.5 × interquartile range. Two-sided Wilcoxon rank-sum tests were used (with *P* values adjusted via the Benjamini–Hochberg procedure to control the FDR) to compare EMP and LMP as well as clusters p1 and p2. **h**, Violin plots depicting prospective LMP fate (top) and EMP fate (bottom) scores across early progenitor clusters p1 and p2 as well as EMP and LMP populations. EMP fate (8 genes) and LMP fate (14 genes) signatures (Supplementary Table 1) were extracted by intersecting pairwise differential gene expression results (EMP versus LMP and P1 versus P2; Extended Data Fig. 3f). Embedded boxplots indicate the median (horizontal line), the upper and lower hinges represent the 75th and 25th percentile and whiskers extend to 1.5 × interquartile range. Two-sided Wilcoxon rank-sum tests were used (with *P* values adjusted via the Benjamini–Hochberg procedure to control the FDR) to compare EMPs and LMPs as well as clusters p1 and p2. Source Data

To isolate and functionally characterize the two extra-embryonic HE populations, we screened our data for cell surface markers suitable for FACS enrichment from wild-type (WT) embryos devoid of fluorescent reporters. This highlighted the previously identified *Lyve1* and *CD24a*, as potential markers for respectively the YS-B and YS-A HE trajectory. *Mcam* (melanoma cell adhesion molecule) was expressed at early stages of both trajectories (Fig. 4a,b and Extended Data Fig. 3b,c). Next, we examined by flow cytometry *Runx1*^RFP^ expression, a strong indicator of HE identity, in extra-embryonic KIT^pos^CD31^pos^LIN^neg^ YS endothelial subpopulations defined by a combination of these markers: LYVE1^neg^CD24^pos^MCAM^pos^, LYVE1^neg^CD24^pos^MCAM^neg^, LYVE1^pos^CD24^neg^MCAM^pos^ and LYVE1^pos^CD24^neg^MCAM^neg^. Within the MCAM^pos^ cell populations, few cells displayed transcription of the *Runx1* locus (RFP 1.5–23%), suggesting limited HE enrichment. In contrast, both MCAM^neg^ populations were highly enriched for cells with an active *Runx1* locus (CD24^pos^LYVE1^neg^MCAM^neg^ 72 – 88% and CD24^neg^LYVE1^pos^MCAM^neg^ 62-67%) (Fig. 4c). Subsequent scRNA-seq of cells in these populations confirmed that these MCAM^neg^ populations are enriched for the extra-embryonic HE (Fig. 4d).

To functionally assess the hematopoietic potential of the two YS HE populations, single cells were sorted, co-cultured on OP9, and evaluated for myeloid (GR1, MAC1/CD11b*)*, erythroid (TER119) and lymphoid (CD19) potential by flow cytometry after 14 days of co-culture. Single cells from both YS HE populations displayed hematopoietic activity regardless of the developmental stage (Fig. 4e). Wells seeded with CD24^pos^ cells contained myeloid, lymphoid and mixed lymphoid/myeloid cells, whereas wells seeded with LYVE1^pos^ cells predominantly gave rise to myeloid, erythroid and erythroid/myeloid cells (Fig. 4f). We also utilized our Runx1b^RFP^ reporter model in conjunction with this HE marker panel to enrich for the least progressed MCAM^pos^ endothelial cells within the YS-A (a1, LYVE1^neg^CD24^pos^MCAM^pos^*Runx1*RFP^pos^) and YS-B (b2, LYVE1^pos^CD24^neg^MCAM^pos^*Runx1*RFP^pos^) trajectories. The hematopoietic potential of both MCAM^pos^ cell populations was lower than that of the respective MCAM^neg^ populations (Fig. 4e and Extended Data Fig. 3d,e), but the hematopoietic identity of the output was similar (Fig. 4f and Extended Data Fig. 3d,e).

Finally, as the emergence of the lymphomyeloid-producing HE clusters a1–a2 and the erythromyeloid-producing HE cluster b2–b3 closely correlated with the emergence of respectively cluster p1 and p2 (Fig. 2e), we investigated whether these two progenitor populations show signs of early LMP or EMP commitment based on previously published EMP and LMP gene signatures (Fig. 4g and Supplementary Table 1). Although these signatures could distinguish p1 (which resembled EMP) from p2 (which resembled LMP) the difference between the two progenitor populations was minimal (Fig. 4g). This prompted us to investigate if we could define a more powerful gene signature to identify early EMP and LMP potential during progenitor emergence. We used the intersection of pairwise differential gene expression analysis (LMP versus EMP and p1 versus p2) to extract prospective EMP fate and LMP fate signatures (Supplementary Table 1 and Extended Data Fig. 3f). These ‘fate’ signatures performed better at assigning p1 to an LMP and p2 to an EMP fate (Fig. 4h), suggesting that these gene signatures could be useful to determine whether early progenitors have an EMP or LMP fate.

Overall, these results demonstrate that the two extra-embryonic HE populations associate with wave 2 EMP and LMP production, a finding consistent with their distinct endothelial identities and temporal abundance^32^. Given these findings, we hereafter named the three different HE populations based on their distinct localizations; HE^AGM^ (clusters c3–c4), HE^YSA^/clusters a1–a2, which are found within the YS arteries, HE^YSP^/clusters b2–b3 which are found within the YS endothelial plexus.

### Identification of a shared common HE signature marked by *Gfi1* and *Mycn*

**Fig. 5:**
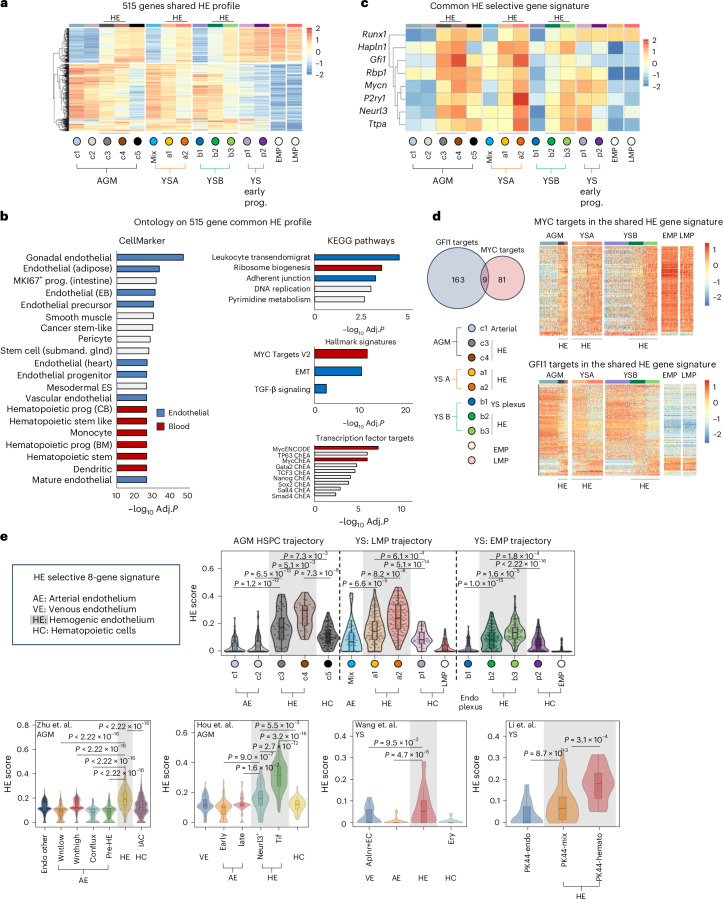
The shared common HE signature is marked by the transcription factors *Gfi1* and *Mycn.* **a**, Heatmap depicting the relative expression of all the 515 genes in the shared common profile across intra- and extra- embryonic EHT trajectories (as defined in Fig. 2b). All HE populations display a mixed expression of genes that are strongly expressed in either the endothelial or hematopoietic arms of the EHT trajectory. YS-derived EMPs and LMPs are included for reference (right). **b**, Gene Ontology analysis of the 515 gene universal HE profile. Top Gene Ontology hits (capped at 20) from the CellMarker, KEGG, Hallmark and ChEA/ENCODE databases are shown. Adjusted *P* values were calculated using Fisher’s exact test with Benjamini–Hochberg correction **c**, Heatmap depicting the relative expression of all seven HE-selective genes within the shared HE profile and *Runx1* across all three EHT trajectories (as defined in Fig. 2b). YS-derived EMPs and LMPs are included for reference (right columns). **d**, The shared HE profile contains many GFI1 and MYCN target genes. Intersect of GFI and MYCN target genes in the universal HE profile (left). Single-cell heatmaps depicting the expression of shared HE profile MYCN target genes (top) and GFI1 target genes (bottom) across intra- and extra- embryonic HE populations (right). **e**, Violin plots demonstrating that the HE signature defined in Fig. 5c effectively identifies HE cells in all three EHT trajectories analyzed in this manuscript (top)^26^ as well as in previously published AGM and YS datasets (bottom)^30,36–38^. Where appropriate the *y* axis of the plots shows the names of the population/cluster nomenclature used in the relevant publications. AE, arterial endothelium; HC, hematopoietic cell. Embedded boxplots indicate the median (horizontal line), the upper and lower hinges represent the 75th and 25th percentile and whiskers extend to 1.5 × the interquartile range. Two-sided Wilcoxon rank-sum tests were used (with *P* values adjusted via the Benjamini–Hochberg procedure to control the FDR) to compare relevant populations. Source Data

**Extended Data Fig. 4:**
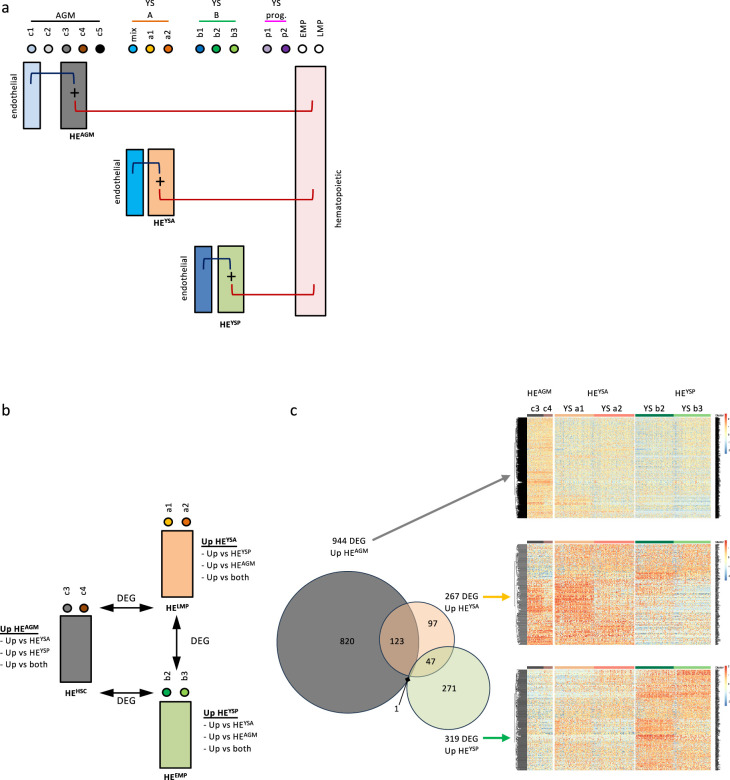
Shared and Unique HE profiles. **a**, Schematic depicting which differential gene expression (DEG) lists (log2FC > 1) were used to establish the shared HE profile. **b**, Schematic depicting how unique HE profiles were established using pairwise comparison between the three HE populations. HE^AGM^ (clusters c3 and c4 from the AGM), HE^YSA^ (clusters a1 and a2 from the YS), HE^YSP^ (clusters b2 and b3 from the YS). **c**, Left: Venn diagram depicting the result of pairwise DEG analysis on HE^AGM^, HE^YSA^ and HE^YSP^. “Up” indicates a gene is upregulated (log2FC > 1.5) vs at least one other HE. The genes present in each group are depicted in the single-cell heatmaps on the right. Genes up in HE^YSP^ show a high overlap with HE^AGM^ gene hits.

Next, we used the three HE transcriptomes to identify shared HE and EHT characteristics. Acknowledging the continuous nature of the EHT process and the hybrid endothelial–hematopoietic identity of the HE, we identified shared differentially expressed genes (DEGs) between the three HE populations and the extremities (non-HE endothelium and the EMP/LMP populations) of the EHT trajectory (Extended Data Fig. 4a and Supplementary Table 2a). The resulting 515 genes profile is a hybrid of genes expressed in endothelial cells (330 of 515) and genes expressed in hematopoietic cells (178 of 515) (Fig. 5a and Supplementary Table 2a). It contains many genes, including *Proc*, *Neurl3*, *Runx1* and *Gfi1* previously associated with an HE identity (Supplementary Table 2a). Ontology analyses revealed enrichments for categories typically associated with HE and EHT including EMT, TGF-β signaling^33^ and ribosome biogenesis (Fig. 5b). Notably, just 7 out of the 515 genes displayed a distinct HE-restricted expression pattern: *Neurl3*, *Hapln1*, *Rbp1*, *Ttpa*, *P2ry1* and the transcription factors *Gfi1* and *Mycn* (Fig. 5c). Almost half (49%, 253 genes) of the genes within the shared profile could be identified as potential targets of these two transcription factors (Fig. 5d and Supplementary Table 2a,b). Most of the potential MYC target genes displayed increased expression toward the hematopoietic end of the three HE. Conversely, most of the GFI1 targets^21,34,35^ were downregulated toward the hematopoietic end (Fig. 5d and Supplementary Table 2a,b). Finally, we verified that the seven HE-restricted genes in combination with *Runx1* can be used as an eight-gene HE-selective gene signature to identify cells with HE characteristics across independent mouse intra-embryonic (AGM)^26,30,36^ and extra-embryonic (YS)^37,38^ scRNA-seq EHT datasets (Fig. 5e).

Overall, we established a shared HE profile that encompasses an eight-gene HE signature that is sufficient to identify cells with HE characteristics. Furthermore, *Gfi1* and *Mycn* are the only transcription factors with a HE-restricted expression pattern.

### Chromatin modifiers and splicing machinery are differentially expressed between intra- and extra-embryonic HE

**Fig. 6:**
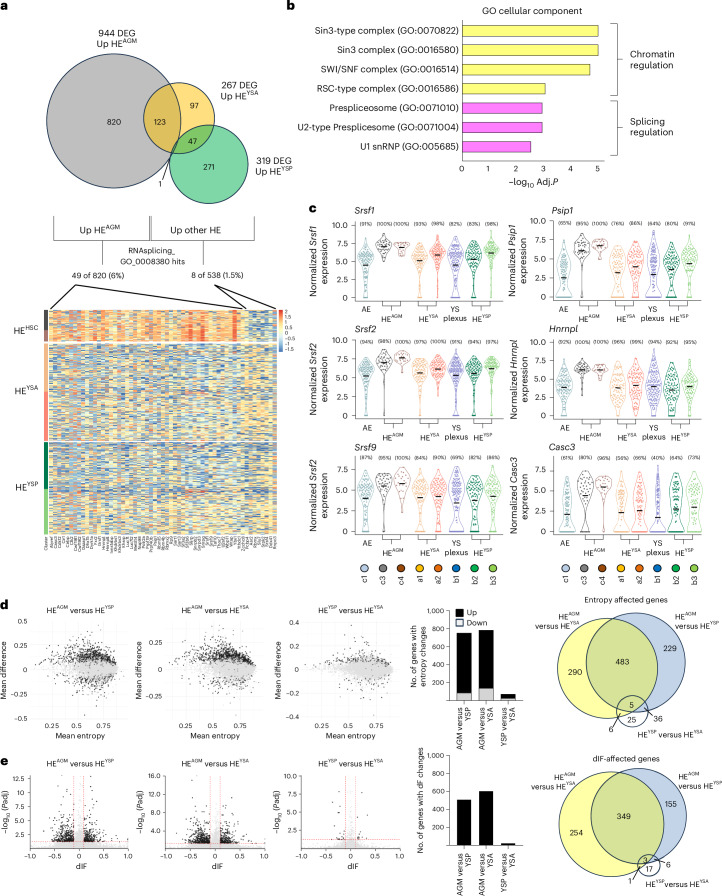
Differential expression of chromatin modifiers and splicing machinery between intra- and extra-embryonic HE correlates with distinct isoform expression landscapes. **a**, Venn diagram depicting the result of pairwise DEG analysis on HE^AGM^, HE^YSA^ and HE^YSP^ (top). ‘Up’ indicates a gene is upregulated (log_2_FC > 1.5) versus at least one other HE population. Single-cell heatmap depicting the genes from GO: RNAsplicing_GO_0008380 that are differentially expressed between the HE populations (bottom). **b**, Gene Ontology analysis (GO cellular components) on genes that are differentially expressed between HE^AGM^ and YS HE (log_2_FC > 1.5 higher expression). Adjusted *P* values were calculated using Fisher’s exact test with Benjamini–Hochberg correction **c**, Violin plots depicting the expression of selected RNA processing genes across HE^AGM^, HE^YSA^ and HE^YSP^. Arterial endothelium and plexus endothelium are shown for reference. **d**, Analyses of isoform entropy difference between HE^AGM^ versus HE^YSP^, HE^AGM^ versus HE^YSA^ and HE^YSP^ versus HE^YSA^. Scatter-plots showing the genes having differential usage pattern for the indicated comparison (left). Black dots represent genes with significant mean entropy differences (mean difference > 0.1, FDR *P*adj < 0.05). Statistical test Wilcoxon signed-rank test, two-tailed. Gray dots represent genes with nonsignificant changes. Bar graphs depicting the number of genes with differential entropy values in the different comparisons (middle). The proportion of genes with increased and decreased entropy values are depicted in black and gray, respectively. HE^AGM^ is skewed toward genes with higher entropy values (chi-squared goodness of fit test, *P* < 0.0001). Venn diagram depicting the intersect of the different comparisons (right). Only a very small set of genes shows entropy differences between the HE^YSB^ versus HE^YSA^. **e**, Differences in isoform fraction (dIF) analyses between HE^AGM^ versus HE^YSP^, HE^AGM^ versus HE^YSA^ and HE^YSP^ versus HE^YSA^. Scatter-plots showing the gene with dIF changes for the indicated comparison (left). Black dots represent genes with significant dIF changes (dIF > 0.1, FDR *P*adj < 0.05). Statistical test used was the IsoformSwitchAnalyzeR implementation of the differential transcript usage (DTU) test in the satuRn R package (https://f1000research.com/articles/10-374/v2) (a generalized linear model-based test). Gray dots represent genes with nonsignificant changes. Bar graphs depicting the number of genes with dIF changes in the different comparisons (middle). Venn diagram depicting the intersect of the different comparisons (right). Only a very small set of genes show dIF differences between the HE^YSP^ versus HE^YSA^. Source Data

**Extended Data Fig. 5:**
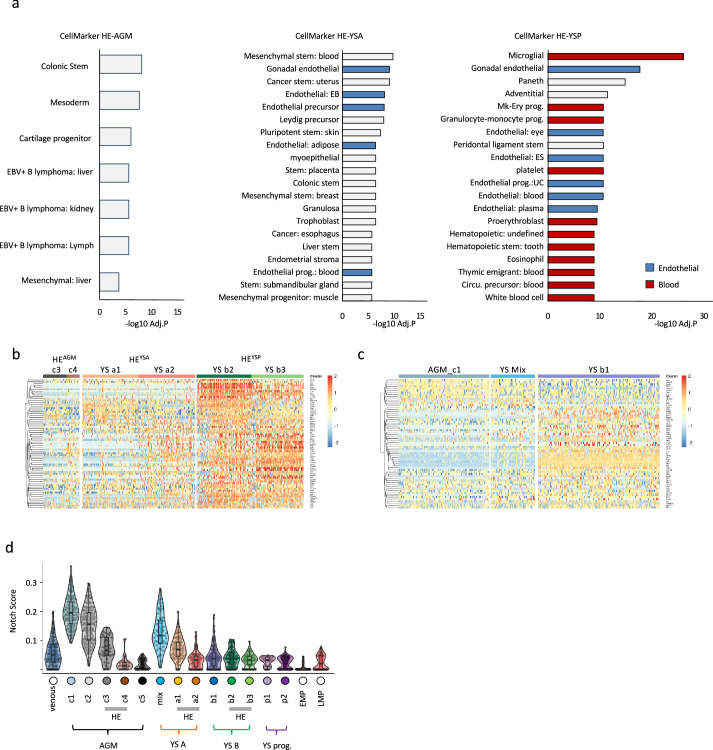
unique HE profiles. **a**, CellMarker analysis on the DEG between the three HE populations. Top hits in the CellMarker database are shown (capped at 20). Genes upregulated in HE^YSP^ have a strong hematopoietic identity. This is not observed in any of the other HE populations. Adjusted p-values were calculated using Fisher’s exact test with Benjamini–Hochberg correction. **b**, Single-cell heatmap of hematopoietic genes, extracted from the HE^YSP^ Cellmarker analysis in (a), across three HE populations. **c**, Single-cell heatmap of hematopoietic gene expression, extracted from the HE^YSP^ CellMarker analysis in (a), across non-HE endothelial populations. Plexus endothelium expresses relatively high levels of hematopoietic genes compared to the other endothelial populations. **d**, Violin plots depicting Notch signature scores across all clusters defined in main Fig. 2b. For reference, AGM-derived venous endothelial cells (left column) and extra-embryonic-derived EMP and LMP populations (right columns) are also included. Embedded boxplots indicate the median (horizontal line), the upper and lower hinges represent the 75^th^ and 25^th^ percentile and whiskers extend to 1.5x the inter-quartile range.

To identify differences between the three HE populations, we conducted pairwise differential expression analyses; HE^AGM^ versus HE^YSA^, HE^AGM^ versus HE^YSP^ and HE^YSA^ versus HE^YSP^ (Fig. 6a, Extended Data Fig. 4b,c and Supplementary Table 3a). Genes significantly upregulated in HE^YSP^ exhibited a distinct (myeloid and erythroid) hematopoietic identity (Extended Data Fig. 5a) with some myeloid genes already expressed within the non-HE YS endothelium (Extended Data Fig. 5b,c and Supplementary Table 3b). Genes significantly upregulated in the other two HE populations did not display a similarly overt hematopoietic signature (Extended Data Fig. 5a and Supplementary Table 3b). HE^YSA^ most closely resembled HE^AGM^ (Extended Data Fig. 4c and Supplementary Table 3a) with the notable expression of Notch pathway components in both the HE^AGM^ and the HE^YSA^ consistent with their arterial identity (Extended Data Fig. 5d)^39^.

**Extended Data Fig. 6:**
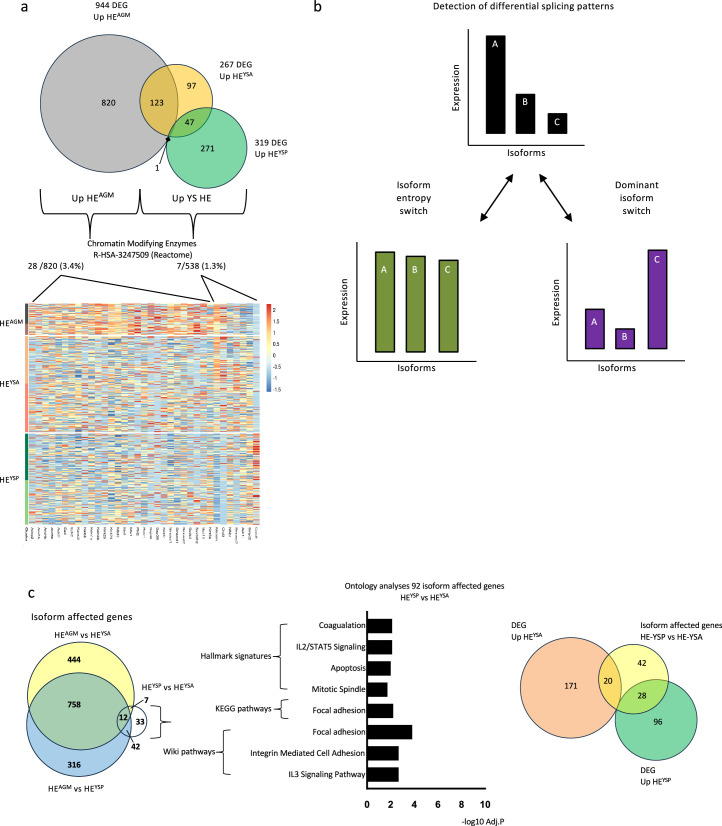
Enrichment of Chromatin modifiers in HE^AGM^ and isoform level analyses of HE populations. **a**, Top: Venn diagram depicting the result of pairwise DEG analysis on HE^AGM^, HE^YSA^ and HE^YSP^. “Up” indicates a gene is upregulated ( > logfc1.5) vs at least one other HE. Bottom single-cell heatmap depicting the genes from Chromatin Modifying Enzymes R-HSA-3247509 (Reactome) that are differentially expressed between the HE populations. **b**, Schematic representation of different isoform usage patterns. Left: schematic representation of isoform Mean Entropy Differences (MED). Right: Schematic representation of dIF (difference in Isoform Fraction) changes **c**, Analyses of isoform differences (entropy and/or dIF) between HE^AGM^ vs HE^YSP^, HE^AGM^ vs HE^YSA^ and HE^YSP^ vs HE^YSA^. Left: Ven diagram depicting the intersect of the different comparisons. Only a small set of 94 genes show isoform level differences between the HE^YSA^ vs HE^YSP^. Middle: Gene ontology analyses across Wikipathways 2024 Mouse, KEGG 2021 human and MSigDB Hallmark 2020 databases. The input gene lists consisted of the 92 genes that showed differential isoform expression (entropy and/or dIF) between HE^YSA^ and HE^YSP^. All significant hits are shown. Gene lists can be interrogated in Supplementary Table 4. Adjusted p-values were calculated using Fisher’s exact test with Benjamini–Hochberg correction. Right: Ven diagram showing the overlap of the isoform affected genes and genes that are differentially expressed between the two YS HE populations (HE^YSA^ and HE^YSP^).

Overall, HE^AGM^ contained a large group of genes that were more highly expressed compared to one or both extra-embryonic HE (Fig. 6a, Extended Data Fig. 4c and Supplementary Table 3a). Gene Ontology analysis identified two main functionalities within these HE^AGM^ selective genes; chromatin modification and RNA processing/splicing (Fig. 6b and Supplementary Table 3a–c). Specifically, 28 genes related to chromatin modification (Extended Data Fig. 6a) and 49 RNA processing genes (Fig. 6a) demonstrated a ≥ 1.5 log_2_ fold change (FC) in HE^AGM^ over at least one of the extra-embryonic HE populations (Supplementary Table 3a–c).

The HE^AGM^-specific upregulation of RNA processing genes is in line with a recent study describing changes in RNA transcript diversity during AGM EHT^40^. Indeed, we observed HE^AGM^-specific upregulation of genes encoding the splice site recognition proteins SRSF1, SRSF2 and SRSF9, implicated in changes in transcript diversity observed during EHT in the AGM^40^ (Fig. 6c and Supplementary Table 3a). The most differentially expressed RNA processing factors included *Psip1*, encoding a SRSF1 interacting protein, *Hnrnpl*, an activator/repressor of exon inclusion, and *Casc3*, which functions in the non-sense-mediated decay pathway (Fig. 6c and Supplementary Table 3c).

Overall, these analyses reveal that the HE^YSP^ has a distinct hematopoietic profile. Furthermore, HE^AGM^ displays a unique gene expression profile, not observed in either YS HE, characterized by higher expression levels of chromatin modifiers and spliceosome components.

### HE^AGM^ transcriptome displays a higher isoform complexity compared to extra-embryonic HE populations

The increased expression level of splicing-related genes in HE^AGM^ suggests that this HE has a distinct isoform expression landscape compared to the YS HE populations. To assess this, we queried our Smart-seq2 dataset at the isoform level. To interrogate differences in isoform expression patterns on a gene level we calculated changes in gene entropy (mean Laplace entropy difference) and dIF (difference in isoform fraction) between the three HE populations^41,42^. In this context increased entropy represents a shift toward a higher transcriptome complexity (more balanced expression of multiple isoforms), while dIF calculations detect shifts in the dominant isoform expressed from a given locus (Extended data Fig. 6b). Both these metrics highlighted a prominent difference in the isoform landscape in the AGM HE compared to both YS HE populations (Fig. 6d,e and Supplementary Table 4a–d). In contrast only minor differences were observed in between the two YS HE populations (Fig. 6d–e and Supplementary Table 4a–d). A total of 1,049 gene loci showed significant differences in entropy (Fig. 6d) compared to one or both YS HE populations. Furthermore, the vast majority (84%) of these gene loci showed increased entropy values in HE^AGM^ compared to the YS HE populations. In contrast, entropy differences were found in only 72 gene loci when the two YS HE were compared to each other, with 64% showing increased entropy within the HE^YSP^ when compared to HE^YSA^. Analysis of dIF changes (Fig. 6e) gave similar results with a large set of genes (768) showing significant shifts in dominant isoform expression when comparing HE^AGM^ to one or both YS HE populations. 46% of these genes had different dIF values compared to both HE^YSA^ and HE^YSP^. Only a small set of 27 genes displayed significant dIF differences between the YS HE populations.

**Fig. 7:**
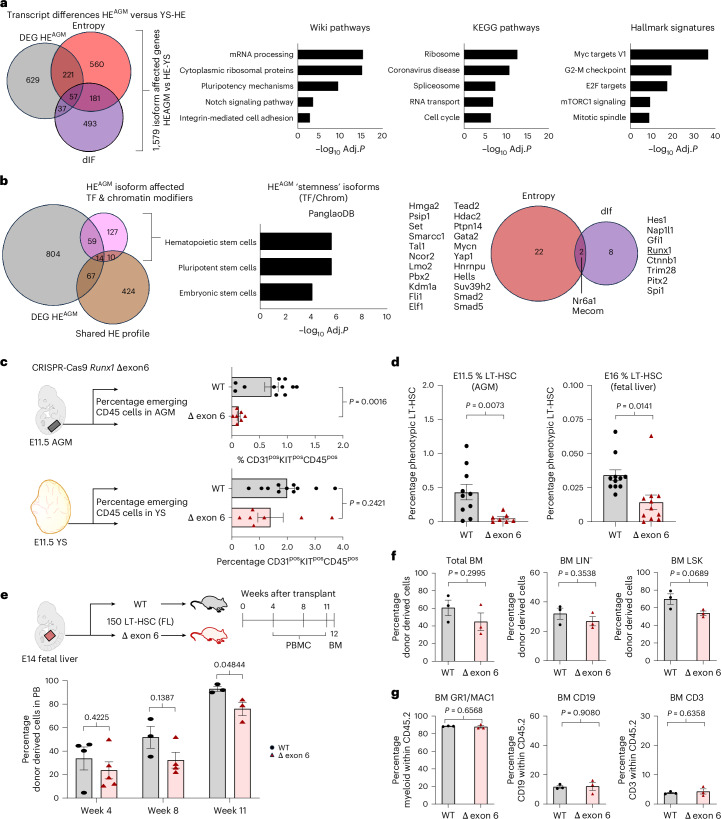
Loss of exon 6 containing Runx1 isoforms impacts HSC emergence. **a**, Venn diagram showing the intersect between isoform-based entropy and dIF level differences between HE^AGM^ and one or both extra-embryonic HE populations as well genes found to upregulated in the HE^AGM^ (as shown in Fig. 6a) (left). Gene Ontology analyses across Wikipathways 2024 Mouse, KEGG 2021 human and MSigDB Hallmark 2020 databases (right). The input gene lists consisted of the 1,579 genes that showed differential isoform expression (entropy and/or dIF) between HE^AGM^ and one or both YS HE. Gene lists can be interrogated in Supplementary Table 4. Adjusted *P* values were calculated using Fisher’s exact test with Benjamini–Hochberg correction **b**, Venn diagram depicting the intersects between genes upregulated in the HE^AGM^ (as depicted in Fig. 6a), the shared HE profile (as depicted in Fig. 5a) and the 210 gene list of transcription and chromatin factors with distinct HE^AGM^ isoform expression profiles (left). Cell identity analysis performed on all transcription and chromatin factors present in the list of genes with potential HE^HSC^-selective isoform expression (middle). Adjusted *P* values were calculated using Fisher’s exact test with Benjamini–Hochberg correction. Venn diagram intersect for the stemness genes identified in the cell identity analyses (right). The diagram shows if the genes were selected based on changes in Entropy or dIF. **c**, Emerging hematopoietic cells (CD31^pos^KIT^pos^CD45^pos^) in E11.5 WT and CRISPR-Cas9 Runx1 Δ exon 6 embryos identified by flow cytometry (Extended Data Fig. 1d). Percentage of emerging hematopoietic cells in E11.5 AGM regions (top). Each point represents a single AGM. Percentage of emerging hematopoietic cells in E11.5 YSs (bottom). Each point represents a single YS. WT *n* = 11, Δ exon 6 *n* = 7. Bars represent the average percentage of emerging hematopoietic cells ± s.e.m. Statistical test used was an unpaired two-tailed *t*-test. **d**, Phenotypic LT HSCs in E11.5 AGM and E16 FL identified by flow cytometry (Extended Data Fig. 1d). Left, percentage of LT HSCs in E11.5 AGMs. Each point represents a single AGM (WT *n* = 10, Δ exon 6 *n* = 7). Right, percentage of LT HSCs in E16 FLs. Each point represents a single FL (WT *n* = 11, Δ exon 6 *n* = 11). Bars represent the average percentage of LT HSC ± s.e.m. Statistical test used was an unpaired two-tailed *t*-test. **e**, Schematic of E14 FL LT HSCs transplantation experiments (top). A total of 150 phenotypic FL LT HSCs (CD45.2) were transplanted into sublethally irradiated NSGS mice (CD45.1). Donor contribution was followed for 12 weeks. Peripheral blood was analyzed by flow cytometry 4, 8 and 11 weeks post-transplant. At 12 weeks post-transplant the bone marrow (BM) was collected and analyzed by flow cytometry. Contribution of donor cells (CD45.2) to the peripheral blood of the transplanted mice at week 4 (WT *n* = 4, Δ exon 6 *n* = 5), 8 (WT *n* = 3, Δ exon 6 *n* = 4) and week 11 (WT *n* = 3, Δ exon 6 *n* = 3) (bottom). Bars represent the average percentage of donor derived blood cells  ± s.e.m. Unpaired two-tailed *t*-test. **f**, Bar graphs presenting the percentage of donor derived cells in the BM of recipient mice 12 weeks post-transplant. Donor cell contribution to the total BM (left). Donor cell contribution to the lineage negative (TER-119^neg^CD3^neg^B220^neg^GR1^neg^MAC1^neg^) BM population (middle). Donor cell contribution to the BM LSK (TER-119^neg^CD3^neg^B220^neg^GR1^neg^MAC1^neg^SCA1^pos^KITt^pos^) population (right). *n* = 3. Bars represent the average percentage of donor derived blood cells ± s.e.m. Unpaired two-tailed *t*-test. **g**, Myeloid (GR1^pos^ and/or MAC1^pos^), B cell (CD19^pos^) and T cell (CD3^pos^) lineage output of donor cells in recipient BM 12 weeks post-transplant. *n* = 3. Bars represent the average percentage of lineage contribution of donor derived blood cells  ± s.e.m. Unpaired two-tailed *t*-test. Source Data

**Extended Data Fig. 7:**
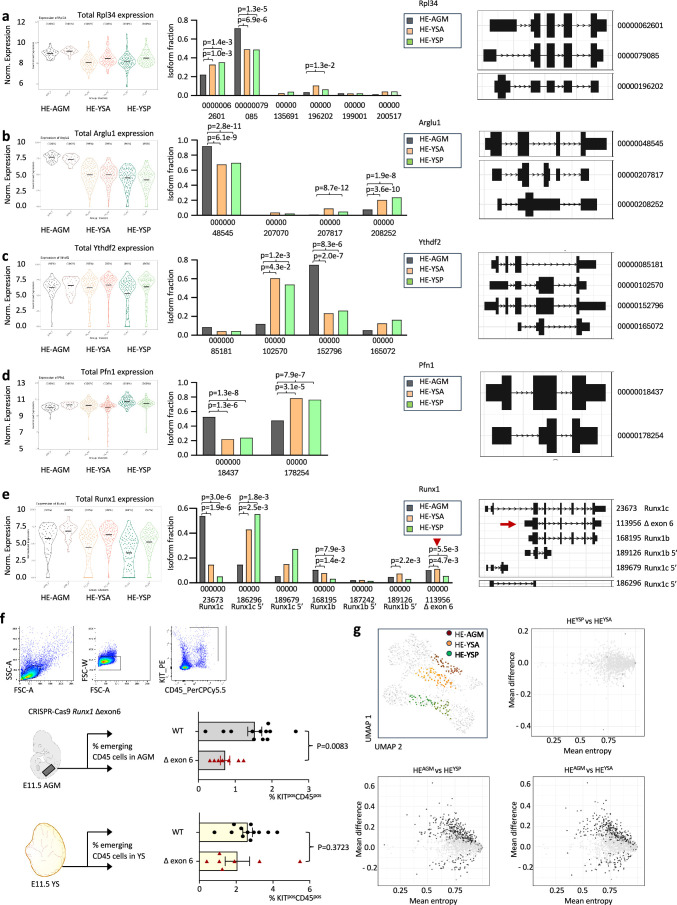
The unique isoform landscape of HE^AGM^. **a-e**, gene and isoform expression for *Rpl34* (**a**), *Arglu1* (**b**), *Ythdf2* (**c**), *Pfn1* (**d**) and *Runx1* (**e**). Left: violin plot of single cell normalized (total) gene expression in HE^AGM^ (clusters c3-c4), HE^YSA^ (clusters a1-a2) and HE^YSP^ (cluster b2-b3), Middle: isoform usage bar graph depicting all detected isoforms (ENSMUST). Statistical test used was the IsoformSwitchAnalyzeR implementation of the DTU test in the satuRn R package [https://f1000research.com/articles/10-374/v2] (a generalized linear model-based test). Right: schematic of relevant isoforms. The red arrow in (**e**) highlights the *Runx1* isoform that lacks exon 6. **f**, Emerging hematopoietic cells (KIT^pos^CD45^pos^) in E11.5 wildtype and CRISPR-Cas9 Runx1 Δ exon 6 embryos identified by flow cytometry. Top, representative analysis flow cytometry plots. Middle, the percentage of KIT^pos^CD45^pos^ cells in E11.5 AGM regions. Each point represents a single AGM. Bottom, the percentage of KIT^pos^CD45^pos^ cells in E11.5 Yolk sacs. Each point represents a single yolk sac. WT N = 12, Δ exon 6 N = 7. Bars represent the average + s.e.m. Statistical test: unpaired two-tailed t-test. **g**, Nanopore long-read sequencing of embryonic HE populations. Top right: single cell UMAP depicting three embryonic EHT trajectories as depicted and described in main Fig. 2a-b. The dots indicate 160 cells that have been re-sequenced on the nanopore long-read platform. Right and bottom: Analyses of isoform entropy difference between HE^YSP^ vs HE^YSA^, HE^AGM^ vs HE^YSP^ and HE^AGM^ vs HE^YSA^ and. Left: scatter plots showing the genes having differential usage pattern for the indicated comparison. Black dots represent genes with significant mean entropy differences (mean difference > 0.1, FDR adjP < 0.05). Statistical test: Wilcoxon signed-rank test, two-tailed. Gray dots represent genes with non-significant changes. Source Data

Collectively, 1,597 genes exhibited isoform level differences between HE^AGM^ and one or both extra-embryonic HE populations. This gene set included a substantial fraction of genes not detected by standard differential gene expression analyses (Fig. 7a left and Supplementary Table 4a), highlighting that the two methods of analyses capture distinct subsets of potential effector genes. Ontology analyses (Fig. 7a right and Supplementary Table 4e) of the set of 1,597 revealed enrichment of genes involved in basal cellular machineries, including RNA (spliceosome), ribosome and cell cycle-related ontologies. Overall, we found that singular clear shifts from one specific isoform to another were rare, with often multiple different (sized) transcripts (both coding and noncoding) showing subtle shifts in proportions (Supplementary Table 4). Examples (Extended Data Fig. 7a–d) were we observed discernible differential splicing events (defined as, partial or complete, exon inclusion/exclusion changes) between HE^AGM^ and YS HE are *Rpl34*, encoding part of the large 60s ribosomal subunit; *Arglu1* a splice modulator; *Ythdf2* a m⁶A-dependent RNA degrader; *Pfn1*, a cytoskeleton modulating protein. Notably, the latter two have been implicated in HSPC biology^43,44^. Ontology analyses of the small number of genes affected on an isoform level between HE^YSP^ and HE^YSM^ did not yield robust Gene Ontology results (Extended Data Fig. 6c and Supplementary Table 4e).

Together, these data demonstrate that the increased expression of splicing-related genes in HE^AGM^ correlates with an isoform expression landscape that is distinct from both YS HE populations.

### Exclusive expression of Runx1 Δ exon 6 transcripts negatively impacts HSC emergence

To focus on potential drivers of an HSC fate we restricted our analyses to transcription/chromatin factors within the HE^AGM^ isoform list (210/1579; Fig. 7b and Supplementary Table 4a). As with the full list, this sublist also contained a substantial number of candidates not found by differential gene expression or analyses of the shared HE profile (Fig. 7b left and Supplementary Table 4a). Cell-type ontology analyses revealed an enrichment for factors associated with stemness (Fig. 7b middle and Supplementary Table 4e). Most of these factors (24 of 32) displayed differential entropy values (Fig. 7b right and Supplementary Table 4a). The majority of the genes in the entropy category displayed increased entropy values in HE^AGM^ (23 of 24), which precludes the identification of a single dominant differentially expressed isoforms (Supplementary Table 4). A small subset of the genes (*n* = 8) demonstrated dIF changes. Further screening for isoform differences between HE^AGM^ and both YS HE populations, as well as differential splicing events, highlighted *Runx1* as a notable candidate.

Multiple annotated *Runx1* transcripts showed shifts in proportion between the three HE populations (Extended Data Fig. 7e). Overall, there is a mix of distal transcripts (Runx1c and short Runx1c 5′ transcripts) and proximal transcripts (Runx1b and short Runx1b 5′ transcripts). The pattern of expression suggests the three populations are at different stages of shifting from the earliest expressed isoform, *Runx1b*, toward the late expressed isoform *Runx1c*. The proportion of full Runx1c transcripts is at its highest in HE^AGM^, is decreased HE^YSA^ and is at its lowest in HE^YSP^ (Extended Data Fig. 7e, middle). The opposite was observed for short Runx1c 5′ transcripts (Extended Data Fig. 7e middle and left). We also observed a small but significant twofold increase in the proportion of Runx1 isoforms lacking exon 6 (*Runx1* Δ6) in HE^AGM^ compared to HE^YSP^ (Extended Data Fig. 7e). The difference between HE^AGM^ and HE^YSA^ was much less obvious suggesting that the shift in *Runx1* Δ6 correlates with an arterial identity of the HE.

Although *Runx1c* is known to be preferentially expressed in HSCs^45^, previous manipulations of Runx1 isoform expression, by enforcing the expression of only *Runx1b* transcripts, did not reveal striking effects on the HSC population^46^; however, the absence of Δ6 transcripts has been previously associated with reduced numbers of HSPCs, including long-term HSCs (LT HSCs), in the bone marrow of adult mice^47^. Together with our observations this suggest that the Δ6 isoform could potentially impact positively on HSC emergence; however, the specific exclusion of Runx1 exon 6 has not been evaluated in vivo. Therefore, we generated homozygote *Runx1*Δ6 embryos by CRISPR-Cas9 mediated deletion in mouse zygotes followed by implantation^48^. Analysis of E11.5 YS and AGM regions of these embryos demonstrated a significant reduction of emerging hematopoietic cells (defined as either CD31^pos^KIT^pos^CD45^pos^ (Fig. 7c) or KIT^pos^CD45^pos^ (Extended Data Fig. 7f)) specifically in the AGM regions of Δ6 embryos, while the YS seemed largely unaffected (Fig. 7c). Furthermore, in Δ6 embryos we observed significantly less phenotypic LT HSCs within both the E11.5 AGM (CD31^pos^SCA^pos^KIT^pos^CD45^pos^EPCR^pos^) as well as the E16.5 fetal liver (FL) (LIN^neg^CD48^neg^SCA^pos^KIT^pos^CD150^pos^) (Fig. 7d). To functionally validate and evaluate the phenotypic Runx1Δ6 FL LT HSCs we performed transplantation experiments (Fig. 7e). Equal numbers of FACS-sorted E14 FL LT HSCs were transplanted (150 LT HSCs per mouse. Figure 7e) into sublethally irradiated mice. Both WT and Runx1Δ6 FL LT HSCs were able to reconstitute hematopoiesis in recipient mice; however, Runx1Δ6 LT HSCs exhibited signs of reduced capacity and/or fitness as the contribution to peripheral blood at 11 weeks was slightly lower (*P* < 0.05) (Fig. 7e). Although not statistically significant a similar trend was observed when analyzing week 12 donor contribution in the total bone marrow of the recipient mice, the bone-marrow lineage negative population (Ter119^neg^CD3^neg^B220^neg^GR1^neg^MAC1^neg^) and the bone-marrow LSK (Ter119^neg^CD3^neg^B220^neg^GR1^neg^MAC1^neg^SCA1^pos^KIT^pos^) population (Fig. 7f). Lineage commitment appeared unaffected, with the WT and Runx1Δ6 transplants showing comparable contributions to myeloid (GR1^pos^ and/or MAC1^pos^), CD19^pos^ (B cell) and CD3^pos^ (T cell) populations (Fig. 7g).

Overall, these data indicate that loss of exon 6-containing Runx1 transcripts negatively affects early HSC emergence in the embryo and suggest a balance between exon 6 containing and exon 6 skipping *Runx1* transcripts is required for HSC emergence in the AGM.

## Discussion

Hematopoietic cell therapies are potent treatment modalities for many blood diseases, including cancer. A major bottleneck for these treatments is sourcing sufficient patient compatible blood cells. Consequently, unraveling the molecular cues driving the generation of specific blood cell types, to reproduce these processes in vitro, is of great interest. HSCs and lineage-restricted EMP/LMPs are first established from HE cells during embryogenesis. Intra-embryonic (dorsal aorta) HE, with HSC potential, has been the focus of multiple scRNA-seq studies. Single-cell transcriptomics of HE in the YS, the initial site of EMP and LMP generation, has so far garnered much less attention. Here we present a comprehensive full-length scRNA-seq dataset that covers three parallel embryonic EHT trajectories, each of which contains their own distinct HE population, skewed toward, respectively, HSPCs (intra-embryonic HE^AGM^), LMPs (YS HE^YSA^) and EMPs (YS HE^YSP^).

In contrast to the exclusively arterial identity of intra-embryonic HE^27,49^, extra-embryonic HE activity^50–52,32^ has been reported throughout the YS endothelium, with an arterial identity being linked to LMP generation^32,50,53^. Combining functional assays, whole-mount imaging and scRNA-seq, we reveal the presence of two different YS HE populations with divergent trajectories, one with an arterial endothelial identity and localization (HE^YSA^) and another with a plexus endothelial identity and localization (HE^YSP^). In line with the sequential emergence of embryonic EMPs and LMPs^1^, the two YS HE populations also arise sequentially. Recently, EHT events within the large intra- and extra-embryonic arteries have been linked to the generation of short-term fetal-restricted HSPCs^54^. The ability to enrich HE^LMP^, using our newly identified CD24-LYVE1-MCAM antibody panel, warrants further characterization of the potential of this HE population.

Comparative analysis of all three EHT trajectories identified a shared HE signature composed of a small set of eight genes that can ascertain cells with HE characteristics regardless of their site of origin. Notably, transcriptional repressor *Gfi1* and transcriptional activator *Mycn* were the only two transcription factors with HE-selective expression patterns. *Gfi1* is an established player in EHT initiation via its ability to downregulate the endothelial program^20,21^ as also reflected in our data. Conversely, many genes that were newly activated (upregulated versus endothelium) within the HE populations were MYC target genes. It has been recently shown that, within the context of the AGM, *Mycn* expression is required for efficient EHT^55^. Our data suggest that *Mycn*, like *Gfi1*, is a HE-specific core functional component of EHT progression independent of the embryonic localization. Overall, many genes previously only reported/characterized in the context of AGM EHT, and suggested to be associated with the acquisition of HSPC potential, are also part of our universal HE profile, highlighting the need for comparative analyses to identify specific cell fate regulators.

Probing differential gene expression between HE populations revealed a high degree of similarity between YS HE^YSA^ and intra-embryonic HE^AGM^, likely reflecting their shared arterial endothelial identity. In contrast, YS HE^YSP^ showed a distinct transcriptional profile, characterized by prominent expression of myeloid/hematopoietic genes, which was also partially observed in non-HE endothelial cells of the YS plexus. In HE^AGM^, we found a prominent enrichment of genes involved in chromatin modification and RNA processing. The latter is especially interesting as several recent studies have indicated that changes in the isoform landscape play a role in the emergence of the hematopoietic system and HSC biology^40,56^. One study by Wang et al. focusing on EHT in the AGM has shown distinct changes in isoform expression profiles in the transition toward HE and subsequently T1-preHSCs^40^. The affected genes were involved in RNA metabolic processes, including RNA splicing, RNA transport and ribonucleoprotein complex biogenesis^40^. Our findings are consistent with these observations and further highlight this phenomenon as specific to HE^AGM^. In contrast, differences in the isoform landscapes between the two YS HE populations were minimal, suggesting that their identity and potential are predominantly driven by differential gene expression. Notably, we observed HE^AGM^ specific changes in isoform expression patterns for transcription/chromatin factors, including *Runx1*, associated with a stem cell identity. We experimentally demonstrated here that limiting the isoform diversity of Runx1, by introducing an in-frame deletion of exon 6 in all transcripts, negatively impacted immunophenotypic LT HSC detection in AGM and FL. Of note, the absence of Δ6 transcripts has previously been shown to negatively impact HSPCs, including LT HSCs, in the bone marrow of adult mice^47^. Indeed, it has been reported that Runx1Δ6 isoforms can enhance the transactivation ability of the exon 6 containing Runx1 isoforms in vitro^47^. More recently, the interaction between the ETS factor ELF1 and RUNX1 has been shown to enhance HSC self-renewal and prevent HSC differentiation^57^. Notably, the RUNX1 E26 transformation-specific (ETS) factor binding domain has been localized to the region encompassing *Runx1* exon 6 and exon 7 (ref. ^58^). Together, these data point toward a role for balanced expression of Runx1_exon6 and Runx1_Δexon 6 transcripts in lineage choice.

The dataset presented here provides a unique resource for further characterization of the three HE populations in the mouse embryo. A particularly intriguing observation is that HE^AGM^ exhibits a distinct isoform landscape compared to the YS HE populations; however, identifying isoform combinations that directly determine cell fate remains a substantial challenge. This not only due to the underlying biology, such as the higher isoform entropy observed in HE^AGM^, but also due to technical limitations in isoform resolution from short-read Smart-seq2 data. To attempt to address this, we performed long-read nanopore sequencing on a subset of 220 (160 HE and 60 early progenitors) cells from our Smart-seq2 dataset. While this approach confirmed a global shift toward higher isoform entropy in HE^AGM^ (Extended Data Fig. 7g), the coverage achieved was lower than that of the Smart-seq2 data and insufficient for robust isoform-level analysis. Further advancements in the sequencing depth and accuracy of long-read single-cell technologies will be particularly beneficial for isoform quantification, transcript coverage and the discovery of novel isoforms. Another limitation of our study is the difficulty in reliably predicting the fate of individual transient HE cells at the single-cell level. For example, within the HE^AGM^ population, we cannot tell which cells will become HSC versus other progenitors. Likewise, in HE^YSP^ and HE^YSA^ populations, we cannot predict erythroid versus myeloid or lymphoid versus myeloid outcomes. Current transcriptomic comparisons using predefined signatures (HSCs, EMPs and LMPs) lack the resolution to detect lineage commitment this early in hematopoietic emergence^26^. These early transient cell states, which may disappear before cells become committed progeny, likely influence fate decisions. This underscores the need to compare HE populations with different developmental outcomes, not just committed populations. As discussed above, many ‘AGM-specific’ EHT genes associated with HSPC potential are expressed in multiple HE subsets, indicating a role in EHT rather than in lineage commitment. Identifying and validating these transient states will require improved perturbation screens, lineage tracing, and novel analytical approaches.

To conclude, our results reveal three distinct EHT trajectories and suggest that hematopoietic fate decisions in HE^AGM^, including those toward an HSC cell fate, could at least in part be governed on an isoform level. Overall, our scRNA-seq dataset capturing three distinct EHT trajectories, giving rise to EMPs, LMPs and HSPCs, represents a powerful and unique resource for future investigations of cell fate decision in different HE.

## Methods

### Mouse embryo generation and processing

Mouse work was performed in accordance with the United Kingdom Animal Scientific Procedures Act (ASPA) 1986. Animal experiments performed at the Cancer Research United Kingdom Manchester Institute (CRUK-MI) were approved by the Animal Welfare and Ethics Review Body of the CRUK-MI. Experiments performed at the University of Oxford were approved by the Oxford Clinical Medicine Ethical Review Committee. Mice were housed in individually ventilated cages under standard conditions, including a 12-h light–dark cycle, ambient temperature of 19–23 °C and relative humidity of 45–65%, in accordance with UK Home Office guidelines and institutional protocols. The transgenic reporter mouse lines (strain C57BL/6JOlaHsd) *Gfi1* (refs. ^20,26,29^) and *Runx1b*^RFP^ (refs. ^26,28^) and *Vwf*^eGFP^ (ref. ^31^) have been described previously. Vaginal plug detection was considered as E0.5 and staging was confirmed for each embryo at the time of collection by visual inspection. For experiments using the *Gfi1*^GFP^ and *Runx1b*^RFP^ reporters, WT Hsd:ICR (CD-1) females were used to set up breeding pairs ensuring reporter sorted cells were exclusively obtained from embryos. The following primers (custom DNA Oligos Merck) were used to genotype embryos. Gfi1GFP, forward1_5′-CCCTTCTCTCAGAACTCAGAG-3′, forward2_5′-GGAAACGAGGTGGCTTGGAG-3′, reverse_5′-GTCTTGTAGTTGCCGTCGTC-3′ (WT: 245 bp, KI:390 bp). Runx1bRFP, forward1_5′-ATGGTGATACAAGGGACATCTTCCC-3′, forward2_5′-ACTTGTATGTTGGTCTCCCG-3′, reverse_5′-ACCAGAGACTTCTACTACAGGC-3′ (WT, 550 bp; KI, 200 bp).

For the single cell RNA-seq and in vitro functional assays, dissected YSs were digested in a mix of Collagenase IV (2 mg ml^−1^, Worthington) and DNase I (200 U ml^−1^, Calbiochem) at 37 °C for 15 min. The dissociated cells were pelleted (300*g* for 5 min at 4 °C) and resuspended in phosphate buffered saline (PBS) containing 10% fetal bovine serum (FBS) and further processed for FACS analyses/sorting.

For YS preparation for whole-mount immunofluorescence staining^25,59,60^, embryos were dissected in calcium and magnesium-free PBS, 10% FBS and 0.1 mM EDTA. Embryos were fixed in PBS 4% paraformaldehyde for 1 h, rinsed with PBS (3×, 5 min at RT) and incubated in 50% methanol in PBS (4 °C for 10 min). Samples were stored at –20 °C in 100% methanol until further use.

### CRISPR-Cas9 *Runx1* Δ exon 6 embryos

One-cell-stage embryos were electroporated with guides targeting the exon 6 of the *Runx1* gene and Cas9 protein, then reimplanted into surrogate mothers^48^. The guides targeted the following sequences flanking exon 6 of *Runx1* (PAM sequences are underscored) (custom DNA Oligos Merck): 5′-CCTCCCGGTCCCTACACTAGGAC–3′ and 5′-CCCACGGAGCCCACTACCCTCTG-3′ At E11.5, embryos were collected and genotyped using primer pairs flanking exon 6: forward1_5′- AGTGGGCTGAAGGAACCT -3′, reverse1_5′-ACGGATTACAGTCTCCAGGA -3′ (WT 779 bp, ko 539 bp) and forward2_5′ CAAGGGGCAATGTCCAACAA -3′, reverse2 5′- ACCTGGAACCGATAACTGCA -3′ (WT 637 bp, ko 397 bp). The AGMs of these embryos were subsequently dissected and processed and analyzed by flow cytometry to identify any defects in blood cell development^26^. For E16.5 FLs, dissected livers were crushed with the end of a 1-ml syringe through a 40-μm cell strainer into IMDM + 10% FBS.

### Transplantation assay of Runx1 Δ exon 6 embryos

Female NSGS (NOD.Cg-Prkdcscid Il2rgtm1Wjl Tg(CMV-IL3,CSF2,KITLG)1Eav/MloySzJ) (CD45.1) mice, aged 8–12 weeks, were used as recipients after two rounds of irradiation at 200 cGy, 3 h apart. Runx1 Δ exon 6 heterozygote males and females (CD45.2) between the ages of 2–6 months were mated, and vaginal plug detection was considered as day 0.5. E14.5 FLs were genotyped and processed for FACS isolation of LT HSCs (TER-119^neg^CD3^neg^B220^neg^GR1^neg^CD48^neg^SCA1^pos^KIT^pos^CD150^pos^) as described above (Supplementary Table 5 lists the antibodies used). Each recipient received 150 LT HSCs intravenously in 200 μl of PBS, along with 20,000 nucleated bone- marrow cells from NSGS donors as a support. Peripheral blood was taken in weeks 4, 8 and 11 after transplantation and terminal samples were collected in week 12.

### Flow cytometry

Flow cytometry analyses were performed on a BD LSRFortessa X-20 Cell Analyzer (BD Biosciences) and a Novocyte Quanteon (Agilent). All cell sorting was performed on a BD FACSAria III Cell Sorter (BD Biosciences). Antibodies used for FACS are listed in Supplementary Table 5. For scRNA-seq cells were directly sorted into lysis buffer and snap-frozen before further processing. FlowJo software (BD Biosciences) was used to analyze all FACS data.

### In vitro single-cell assays

All single cell assays on YS-derived cells were performed using co-culture with OP9 stromal cells (mouse bone-marrow stromal cell line; ATCC CRL-2749 obtained from the American Type Culture Collection)^21^. In brief, hematopoietic activity assays were performed by FACS sorting single YS cells onto OP9 cells in 96-well plates (one cell per well). The cells were cultured in IMDM (Invitrogen), 10% fetal calf serum, L-glutamine (4 mM), penicillin–streptomycin (50 U ml^−1^), α-monothioglycerol (15 mM), ascorbic acid (50 ng ml^−1^), transferrin (180 μg ml^−1^), IL-11 (5 ng ml^−1^), EPO (2 U ml^−1^), oncostatin M (10 ng ml^−1^), IL-6 (20 ng ml^−1^), bFGF (10 ng ml^−1^), IL-3 (100 ng ml^−1^), SCF (100 ng ml^−1^), Flt3L (100 ng ml^−1^) and 2% leukemia inhibitory factor (LIF) supernatant for 10 days before microscopically scoring wells that showed signs of hematopoietic proliferation.

Hematopoietic lineage potential assays were performed similarly but with a different media composition: αMEM (Invitrogen), 10% fetal calf serum, L-glutamine (4 mM), penicillin–streptomycin (50 U ml^−1^), 2-mercaptoethanol (100 mM), SCF (5 ng ml^−1^), IL-7 (2 ng ml^−1^) and Flt3L (5 ng ml^−1^). After 7 days, wells containing proliferating cells were passaged onto fresh OP9 cells and culture for 7 additional days. Lineages of the hematopoietic cells were defined based on FACS analyses of CD19, CD11b and TER119 cell surface expression (Supplementary Table 5).

### Hematopoietic colony-forming unit assays

FACS-sorted YS and FL populations were examined by culturing cells, with (YS) or without (YS and FL) previous co-culture on OP9 for 48 h, in a semi-solid methylcellulose matrix (MethoCult GF M3434, Stem Cell Technologies). Colony output was determined after 7–10 days of culture by colony morphology. Where applicable, OP9 co-culture was performed in IMDM (Invitrogen), 10% fetal calf serum, L-glutamine (4 mM), penicillin–streptomycin (50 U ml^−1^), α-monothioglycerol (15 mM), ascorbic acid (50 ng ml^−1^), transferrin (180 μg ml^−1^), IL-11 (5 ng ml^−1^), EPO (2 U ml^−1^), oncostatin M (10 ng ml^−1^), IL-6 (20 ng ml^−1^), bFGF (10 ng ml^−1^), IL-3 (100 ng ml^−1^), SCF (100 ng ml^−1^), Flt3L (100 ng ml^−1^) and 2% LIF supernatant.

### Whole-mount Immunofluorescence staining and analyses

Whole-mount staining and analyses^25,59,60^. All primary and secondary antibodies used for immunofluorescence are listed in Supplementary Table 5. Fixed (4% paraformaldehyde) samples were routinely stored at −20 °C in 100% methanol (see ‘mouse embryo generation and processing’ section). Following rehydration, YS samples were treated with a permeabilizing blocking solution (0.2% Triton X-100, 2% donkey serum and 2% FBS) and incubated overnight with primary antibodies. The next day a second step with secondary antibodies was carried out. After staining, YSs were cleared overnight in a 50% solution of glycerol in PBS at 4 °C and then flat-mounted on Superfrost glass slides. Samples were imaged using a Zeiss 710 confocal microscope equipped with a LD LCI Plan-Apochromat ×25/0.8 Imm Corr DIC M27 objective or an EC Plan-Neofluar ×40/1.30 Oil DIC M27 objective. Confocal image acquisition was carried out using Zeiss Zen software v.2.3 SP1; image processing and analysis was carried out using IMARIS Viewer software v.9.7.2 (Bitplane), ImageJ/Fiji (v.2.3.5–2.9.0) and Adobe Photoshop CC 2021. vWF-associated and Lyve1-associated mean of fluorescence intensity (MFI) was measured by ImageJ/Fiji as mean of gray value in a selected area (an example is shown in Fig. 3a) and expressed in arbitrary units. The ratio of vWF-associated MFI to Lyve1-associated MFI was calculated for the same area. Cell counts were performed using Fiji/ImageJ Cell Counter tool.

### scRNA-seq and data processing

Single cells were sorted into wells of a 384-well plate containing lysis buffer and snap-frozen. Libraries were prepared using a modified Smart-seq2 protocol^61^. Paired-end 38 bp or 75 bp sequencing was carried out on the NextSeq500 or NovaSeq 6000 platform (Illumina). Following sequencing, the raw fastq files were obtained by bcltoFastq conversion (v.2.20.0.422) and were subsequently aligned to the mm10 reference genome using STAR aligner (v.2.7.9a) with the argument ‘STARsolo’. This argument allowed simultaneous mapping of reads and quantification of gene expression. The reference genome and gene transfer format file were downloaded from 10x Genomics webpage at https://cf.10xgenomics.com/supp/cell-exp/refdata-gex-mm10-2020-A.tar.gz. The output of ‘STARsolo’ was loaded into R (v.4.1.0) using the Bioconducter package DropletUtils (v.1.12.1). Downstream analyses were conducted in R using SingleCellExperiment (v.1.14.1) and Seurat (v.4.0.6). A total of 2,365 cells was sequenced (795 on the NextSeq500 and 1570 on the NovaSeq 6000). Next, cells with <2,000 detected genes, >15% mitochondrial content and >10% hemoglobin percentage were excluded, leaving 2,255 high quality cells (705 on the NextSeq500 and 1,550 on the NovaSeq 6000). This filtering process was adopted as previously described^26^.

### Analysis of YS EHT scRNA-seq datasets

A total of 1,469 scRNA-seq cells were considered high quality YS cells (115 on the NextSeq5000 and 1,354 on the NovaSeq). During the sequencing, 225 technical replicates (the same cells sequenced twice) were introduced. Duplicated technical replicates were removed, retaining cells with that yielded the highest number of genes leaving a total of 1,214 YS cells. Following read quantification and filtering, single cell analysis was performed using the scater (v.1.20.1) package. Raw counts were log-normalized (logNormCounts), gene variance was modeled (modelGeneVar) and the top 2,000 highly variable genes (HVGs) were identified (getTopHVGs). Following normalization, cells were subsequently clustered using graph-based clustering (buildSNNGraph, parameters: *k* = 10, use.dimred = ‘PCA’). Based on graph-based clustering, we noted groups of outlier cells that (1) contained high percentage of ribosomal genes with the lowest genes detected; (2) contained high expression of hemoglobin gene (*Hbb-y*); (3) were potential mesenchymal cells with high expression of mesenchymal genes (*Dlk1* and *Ptn*); (4) were a distinct cluster of cells expressing the marker *Folr1*; and (5) were matured megakaryocyte or platelet-contaminating cells with high expression Pf4, Gp5 and *Gp5*. These outlier cells (*n* = 139) were excluded leaving 1,075 YS cells. We next used unsupervised hierarchical clustering (hclust) utilizing the ‘ward.D2’ distance measure to cluster the cells. The number of clusters were determined based on the dynamic tree cut functionally (cutreeDynamic) yielded six clusters.

### Integration of AGM and YS EHT scRNA-seq datasets

AGM datasets were obtained from the Gene Expression Omnibus (GEO) (GSE150412)^26^. From the raw fastq sequencing files, we used the same processing pipeline as was used in the YS EHT scRNA-seq (as described above) to obtain sequencing counts in the AGM dataset. The raw counts of the AGM and the raw counts of YS data were jointly analyzed as a single AnnData object using the scanpy workflow (v.1.6.1). Low-quality cells were removed as previously described^26^ and using the same criteria described above. As the previous AGM scRNA-seq cells were sequenced on the NextSeq500 platform and the YS scRNA-seq cells were sequenced on the NovaSeq, a number of AGM FACS-ENDO (*n* = 21) were concurrently isolated, processed and sequenced on the NovaSeq platform with the YS FACS population. Two strategies were employed to determine and subsequently mitigate batch effects. First, differential expression was performed between the FACS-ENDO populations sequenced across the two platforms. Genes with greater than log_2_FC of 1.5 and adjusted *P* value < 0.01 were considered as genes associated with experimental batches. Second, gene that showed variation in detection rates (>50%) between the sequencing platform were identified. These genes were excluded from further analysis.

To focus on the similarity and differences during EHT in the AGM and YS, the non-EHT related populations (AGM venous endothelial and AGM mesenchymal) and the YS FACS-HE^KIT-Neg^ cells, YS clusters that have progressed beyond the early progenitor stage (YS EMP and YS LMP) were computationally excluded. The raw counts of the remaining cells were log-normalized (sc.pp.normalize_total) and HVGs identified (sc.pp.highly_variable_genes). To generate low dimensional representation, principal-component analysis (PCA) (sc.tl.pca) was conducted on the scaled expression values (sc.pp.scale). The top 50 principal components were used to determine a *k*-nearest neighbor graph (sc.pp.neighbors(n_neighbors = 20)). Two rounds of semi-supervised Leiden clustering were carried out to identify clusters. Initially, an unsupervised Leiden clustering (sc.tl.leiden(resolution = 2)) was used followed by a semi-supervised merging of clusters with <40 cells. Next, to generate a simplified graph representation of the data, partition-based graph abstraction (PAGA) (sc.pl.paga) was used based on the Leiden groupings. The final UMAP representation was generated using PAGA-initialized positions. The scanpy results were imported into R, where the final representations of the data were generated.

### Differential expression analysis and construction of a common HE signature

Differential expression between two groups was performed using the ‘limma’ package (v.3.54.2) and the ‘voom’ function. Before differential expression, genes with more than 90% dropout were excluded. Additionally, to mitigate skewing of differential gene expression analyses between HE groups in the HE^AGM^, HE^YSA^ and HE^YSP^ trajectories, HE clusters were downsampled to the cluster with the lowest cell number in each individual HE. This resulted in three normalized HE populations, each encompassing two clusters with equal representation. The normalized HE was used to generate a universal HE gene expression profile by performing differential expression analyses versus the closest endothelial population and versus the most progressed hematopoietic cells in our dataset (EMPs and LMPs) as illustrated in Extended Data Fig. 3a. Only genes expressed in at least 33% of the cells (for each type of HE) that displayed a log_2_FC > 1 and false discovery rate (FDR) < 0.05 versus either the endothelial or hematopoietic ends of the trajectory were taken forward. For inter-HE differential gene expression, the following cut-offs were used: log_2_FC > 1.5, FDR < 0.05 and percent of gene-expressing cells (in the upregulated population) >50%. Differentially expressed gene lists were interrogated for enrichment of biological features using the online Enrichr tool^62^. Only results with an adjusted *P* value < 0.05, an odds ratio >2 and >5 gene hits were taken forward.

### Calculation of gene signature scores

To determine a collective gene signature enrichment, the UCell package^63^ (v.2.2.0) was used. Based on a given gene list, the UCell signature score (ScoreSignatures_UCell) was calculated for each cell. Gene list for the different signatures used are listed in Supplementary Table 1. EMP-fate and LMP-fate signature were constructed by intersecting the DEGs between EMPs and LMPs (adjusted *P* value < 0.05) and YS clusters p1 and p2 (adjusted *P* value < 0.05).

#### Statistical comparison of UCell scores

To compare UCell signature enrichment between cell clusters, pairwise statistical comparisons were performed using the Wilcoxon rank-sum test (Mann–Whitney *U*-test). This nonparametric test was chosen due to the non-normal distribution of UCell scores, which typically exhibit right-skewed distributions with a high proportion of zero values. Statistical significance was assessed at *P* = 0.05, and comparisons were visualized using the ggsignif package. For analyses involving multiple comparisons, *P* values were adjusted using the Benjamini–Hochberg method to control the FDR.

##### Analysis of publicly available scRNA-seq data

We analyzed the published scRNA-seq data of Fadlullah et al.^26^, Zhu et al.^30^, Hou et al.^36^, Wang et al.^38^ and Li et al.^37^. In the scRNA-seq data from Fadlullah et al.^26^, we reprocessed the data from raw fastq files using the STARsolo workflow described above. We extracted cluster information and retained the following AGM EHT population: Arterial endothelial, Pre-HE, HE-HSC, and IAHC. In the scRNA-seq data from Zhu et al.^30^, we directly downloaded the count matrix files and the cell annotations from GEO (GSE137116). The Zhu et al. data were filtered to retain cells from E10.5 embryos. Furthermore, only cells from the populations related to EHT were kept: ‘Endo (other)’, ‘Endo (Wnt_low) [AE]’, ’Endo (Wnt_high) [AE]’, ‘Conflux endo [AE]’, ‘Pre-HE [AE]’, ‘HE’ and ‘IAC’. In the scRNA-seq data from Hou et al. (GSE139389), we downloaded the count matrix files from GEO and extracted cluster annotations from the supplementary data (sheet 8, 41422_2020_300_MOESM5_ESM.xls). We retained AGM E10.0-E10.5 endothelial cells corresponding to the following populations: ‘vECs’ (venous endothelial cells), ‘earlyAEC’ (early arterial endothelial cells), ‘lateAEC’ (late arterial endothelial cells), ‘Neurl3-EGFP^+^’ (Neurl3-positive cells), ‘tif-HEC’ (transcriptomic and immunophenotypic and functional HEC) and ‘HC’ (hematopoietic cells). In the scRNA-seq data from Wang et al. (GSE167588), we downloaded count matrix files from GEO and extracted cluster annotations from the supplementary data (11427_2021_1935_MOESM7_ESM.xls). The dataset included both YS and caudal region populations. We retained the YS: ‘YS_Aplnr^+^ EC’ (YS Aplnr-positive endothelial cells), ‘YS_aEC’ (YS arterial endothelial cells), ‘YS_HE’ (YS HE), ‘YS_Ery’ (YS erythroid cells). In the scRNA-seq data from Li et al. (GSE173833), we downloaded count matrix files from GEO (GSM5281418) for YS PK44 (CD41^−^CD43^−^CD45^−^CD31^+^CD201^+^Kit^+^CD44^+^) cells from E10.0 embryos. As cluster annotations were not provided, we performed hierarchical clustering using DEGs between endothelial-biased and hematopoietic-biased populations as described in the original publication (Supplementary Table 1 of the publication). We used Ward’s linkage method with Euclidean distance and dynamic tree cutting to identify three distinct clusters (PK44-endo, PK44-mix and PK44-hematopoetic) representing different stages of YS cell progression.

### Publicly available GFI genomic binding data

The following three GFI binding datasets (GEO accession codes: GSE57251, GSE22178, GSE69101) obtained from early hematopoietic populations were used: (1) GFI1 and GFI1b binding data from DamID of HE from embryonic stem cells, GEO_GSE57251: GSM1377856, GSM1377857 and GSM1377858 (ref. ^21^); (2) GFI1b binding data from ChIP–seq of HPC7 cell line (downloaded from Supplementary Table 1 of the online version of the manuscript). Also available at GEO_GSE22178: GSM552235 and GSM552236 (ref. ^35^); (3) GFI1 and GFI1b binding data from ChIP–seq of mES-derived early hematopoietic progenitors, GEO_GSE69101: GSM1692809, GSM1692853 and GSM1692854 (ref. ^34^). In each of the studies mentioned, the BED files were downloaded and were annotated with ChIPpeakAnno (v.3.20.1). Peaks were filtered to retain regions within 3 kb of transcription start site for ChIP–seq data and 5 kb of the gene body for DamID data. Genes were identified as potential GFI targets if binding was observed in at least one GFI1 and one GFI1b dataset.

### Single-cell isoform transcript analyses

#### SMARTseq

Raw reads were aligned and quantified with Salmon^64^ (v.1.10.2; --libtype OU) against GENCODE transcripts (release 37). Quantification files were read into R using either tximport (v.1.28.0) or importIsoformExpression (IsoformSwitchAnalyzeR v.2.0.1)^42,65^. Isoform switching analysis was perfomed using IsoformSwitchAnalyzeR with isoformSwitchTestSatuRn^42^; significant isoform switches were defined as those with an adjusted *P* ≤ 0.05 and |dIF| > 0.1. Splicing entropy was assessed using SplicingFactory (v.1.8.0)^41^; significant entropy changes were defined with adjusted *P* ≤ 0.05. To detect a skew in entropy changes a chi-squared goodness of fit test was applied.

##### Nanopore sequencing

Data were basecalled using Dorado (v.0.9.1) (Oxford Nanopore Technologies Dorado, 2025; https://github.com/nanoporetech/dorado) using the high-accuracy model (dna_r10.4.1_e8.2_400bps_hac@v5.0.0). Raw reads were trimmed for SMARTseq adapters using bbduk.sh (v.39.08)^66^. Chimeric reads were filtered from the data using YACRD (v.1.0.0) and porechop (v.0.2.4)^67,68^. Cleaned reads were aligned to GENCODE transcripts (release 37) using Minimap2 (v.2.26)^69^, retaining a maximum of ten alignments per read. Alignments were quantified using NanoCount (v.1.0.0.post6)^70^. Abundance files were read into R (v.4.3.0) filtered for low library size samples using findOutliers (scuttle v.1.10.3; type = ‘lower’, nmads = 1) and normalized using DESeq2 (v.1.40.2)^71^. Estimated and normalized counts were subject to analysis using IsoformSwitchAnalyzeR and SplicingFactory as previously described^41,42^.

### Reporting summary

Further information on research design is available in the Nature Portfolio Reporting Summary linked to this article.

## Supplementary information

Reporting Summary

Supplementary Tables Supplementary Tables 1–5, including an inventory of the tables in the first tab.

## Source data

Source Data Figs. 1–7 Statistical source data.

Source Data Extended Data Figs. 2, 3, 7 Statistical source data.

## Data Availability

Gene expression data can be queried at https://shiny.cruk.manchester.ac.uk/AGM_YS_dataset_final/. Raw data are deposited in the GEO under accession codes GSE274544 and GSE309071. Three Source data files accompany this manuscript (for the main figures, extended figures and tables).

## Code availability

Code is available at https://github.com/zakiF/PublishedPapers/tree/master/YolkSac_AGMand https://github.com/RASellers-CRUK/GL_IsoformAnalysis
