# Supplementary material for: Single-cell profiling reveals three endothelial-to-hematopoietic transitions with divergent isoform expression landscapes
Source: Nat Cardiovasc Res. 2025 Nov 11;4(12):1642–61. doi: 10.1038/s44161-025-00740-z (PMC12708354; doi:10.1038/s44161-025-00740-z)
Supplement: Supplementary file 1 — Reporting Summary [file 44161_2025_740_MOESM1_ESM.pdf]

nature portfolio

Corresponding author(s): Michael Lie-A-Ling, Georges Lacaud

Last updated by author(s): Sep 18, 2025

Reporting Summary

Nature Portfolio wishes to improve the reproducibility of the work that we publish. This form provides structure for consistency and transparency in reporting. For further information on Nature Portfolio policies, see our Editorial Policies and the Editorial Policy Checklist.

Statistics

For all statistical analyses, confirm that the following items are present in the figure legend, table legend, main text, or Methods section.

n/a	Confirmed	
☐	☒	The exact sample size (*n*) for each experimental group/condition, given as a discrete number and unit of measurement
☐	☒	A statement on whether measurements were taken from distinct samples or whether the same sample was measured repeatedly
☐	☒	The statistical test(s) used AND whether they are one- or two-sided<br>*Only common tests should be described solely by name; describe more complex techniques in the Methods section.*
☒	☐	A description of all covariates tested
☐	☒	A description of any assumptions or corrections, such as tests of normality and adjustment for multiple comparisons
☐	☒	A full description of the statistical parameters including central tendency (e.g. means) or other basic estimates (e.g. regression coefficient) AND variation (e.g. standard deviation) or associated estimates of uncertainty (e.g. confidence intervals)
☐	☒	For null hypothesis testing, the test statistic (e.g. F, t, r) with confidence intervals, effect sizes, degrees of freedom and P value noted<br>*Give P values as exact values whenever suitable.*
☒	☐	For Bayesian analysis, information on the choice of priors and Markov chain Monte Carlo settings
☐	☒	For hierarchical and complex designs, identification of the appropriate level for tests and full reporting of outcomes
☐	☒	Estimates of effect sizes (e.g. Cohen's *d*, Pearson's *r*), indicating how they were calculated

Our web collection on statistics for biologists contains articles on many of the points above.

Software and code

Policy information about availability of computer code

Data collection	Zeiss Zen software version 2.3 SP1;<br>IMARIS Viewer software version 9.7.2 (Bitplane),<br>ImageJ/Fiji (versions 2.3.5-2.9.0) and Adobe Photoshop 504 CC 2021.<br>Fiji/ImageJ Cell Counter tool<br>FlowJo (v10.1)
Data analysis	Zeiss Zen software version 2.3 SP1;<br>IMARIS Viewer software version 9.7.2 (Bitplane),<br>ImageJ/Fiji (versions 2.3.5-2.9.0) and Adobe Photoshop 504 CC 2021.<br>Fiji/ImageJ Cell Counter tool<br>FlowJo (v10.1)<br>bcltoFastq conversion (version 2.20.0.422)<br>STAR aligner (version 2.7.9a)<br>R (version 4.1.0)<br>Bioconducter package DropletUtils (version 1.12.1).<br>SingleCellExperiment (version 1.14.1)<br>Seurat (version 4.0.6).<br>Scater (version 1.20.1)<br>buildSNNGraph<br>scanpy workflow (version 1.6.1)<br>‘limma’ package (version 3.54.2)

UCell package128 (version 2.2.0)
splice aware aligner Salmon 1.4.0
IsoformSwitchAnalyzeR 2.01.07
SplicingFactory 1.8.0
DESeq2 1.40.2
MinionQC.R 1.4.2131
MinKNOW 23.04.6
Guppy 6.5.7
Minimap2 2.26
IGV Desktop App 2.17.0
ChIPpeakAnno version 3.20.1
https://github.com/zakiF/PublishedPapers/tree/master/YolkSac_AGM
https://github.com/RASellers-CRUK/GL_IsoformAnalysis

For manuscripts utilizing custom algorithms or software that are central to the research but not yet described in published literature, software must be made available to editors and reviewers. We strongly encourage code deposition in a community repository (e.g. GitHub). See the Nature Portfolio guidelines for submitting code & software for further information.

Data

Policy information about availability of data

All manuscripts must include a data availability statement. This statement should provide the following information, where applicable:

- Accession codes, unique identifiers, or web links for publicly available datasets
- A description of any restrictions on data availability
- For clinical datasets or third party data, please ensure that the statement adheres to our policy

The following published datasets were used in this study:
GEO
GSE137116
GSE139389
GSE167588
GSE173833
GSM5281418
GSE57251
GSM1377856
GSM1377857
GSM1377858
GSE22178
GSE69101
GSM1692809
GSM1692853
GSM1692854
GRCm39 M33 mouse reference
GRCm39 M32 mouse reference
mm10 reference

For the data generated in this study:
Gene expression data can be queried at https://shiny.cruk.manchester.ac.uk/AGM_YS_dataset_final/. Raw data is deposited in GEO accession GSE274544 and GSE309071. Three source data files accompany this manuscript
NCVR_2024_09_0957A_extended_source.xlsx, NCVR_2024_09_0957A_main_source.xlsx and NCVR_2024_09_0957A supplemental_tables_1.xlsx

Code used:
https://github.com/zakiF/PublishedPapers/tree/master/YolkSac_AGM and https://github.com/RASellers-CRUK/GL_IsoformAnalysis
https://github.com/RASellers-CRUK/GL_IsoformAnalysis

Research involving human participants, their data, or biological material

Policy information about studies with human participants or human data. See also policy information about sex, gender (identity/presentation), and sexual orientation and race, ethnicity and racism.

Reporting on sex and gender	NA
Reporting on race, ethnicity, or other socially relevant groupings	NA
Population characteristics	NA
Recruitment	NA
Ethics oversight	NA

Note that full information on the approval of the study protocol must also be provided in the manuscript.

Field-specific reporting

Please select the one below that is the best fit for your research. If you are not sure, read the appropriate sections before making your selection.

☒ Life sciences ☐ Behavioural & social sciences ☐ Ecological, evolutionary & environmental sciences

For a reference copy of the document with all sections, see nature.com/documents/nr-reporting-summary-flat.pdf

Life sciences study design

All studies must disclose on these points even when the disclosure is negative.

Sample size	No statistical methods were used to predetermine sample size. For in vivo transplantation experiments, five mice per group were used.
Data exclusions	All replicate data were used for statistical analysis and no data were excluded.
Replication	All data were replicated at least one time (N=2 biological experiments)
Randomization	Samples were randomly allocated into experimental groups and processed in no particular order to minimize batch effects.
Blinding	The investigators were not blinded to group allocation during data collection and analysis.

Reporting for specific materials, systems and methods

We require information from authors about some types of materials, experimental systems and methods used in many studies. Here, indicate whether each material, system or method listed is relevant to your study. If you are not sure if a list item applies to your research, read the appropriate section before selecting a response.

Materials & experimental systems

n/a	Involved in the study	
☐	☒	Antibodies
☐	☒	Eukaryotic cell lines
☒	☐	Palaeontology and archaeology
☐	☒	Animals and other organisms
☒	☐	Clinical data
☒	☐	Dual use research of concern
☒	☐	Plants

Methods

n/a	Involved in the study	
☒	☐	ChIP-seq
☐	☒	Flow cytometry
☒	☐	MRI-based neuroimaging

Antibodies

Antibodies used

Antibody Clone Manufacturer Application dilution Experiment
B220 APC RA3-6B2 eBioscience FACS 1:200 Co-culture readout
B220 eF450 RA3-6B2 eBioscience FACS 1:400 Co-culture readout
B220 FITC RA3-6B2 Invitrogen FACS 1:400 YS lineage staining
B220 Biotin RA3-6B2 eBioscience FACS 1:200 FL HSC staining, FACS sort LT-HSC
CD16/CD32 93 Invitrogen FACS 1:100 Fc blocking
CD16/CD32 AF700 93 eBioscience FACS 1:200 YS EMP/LMP staining
CD19 APC_eF780 eBio1D3 eBioscience FACS 1:200 Co-culture readout
CD19 PE-Cy7 eBio1D3 eBioscience FACS 1:200 terminal BM samples
CD24a PE 30-F1 eBioscience FACS 1:200 YS EHT staining
CD24a PerCPCy5.5 M1/69 BD FACS 1:200 YS EHT staining
CD25 Biotin eBio7D4 eBioscience FACS 1:200 Co-culture readout
CD3 APC 145-2C11 eBioscience FACS 1:200 terminal BM samples
CD3 FITC 145-2C11 eBioscience FACS 1:400 YS lineage staining
CD3 biotin 145-2C11 eBioscience FACS 1:200 FL HSC staining, FACS sort LT-HSC
CD31 PECy7 390 BioLegend FACS 1:200 YS EHT, AGM-HSC staining
CD41 APC MWReg30 eBioscience FACS 1:200 AGM /YS lineage staining
CD41 Biotin MWReg30 eBioscience FACS 1:400 AGM /YS lineage staining
CD41 FITC MWReg30 eBioscience FACS 1:400 AGM /YS lineage staining
CD41 PE MWReg30 eBioscience FACS 1:200 YS EHT staining
CD41 PECy7 MWReg30 eBioscience FACS 1:200 YS EMP/LMP staining
CD45 Biotin 30-F11 Invitrogen FACS 1:400 AGM /YS lineage staining
CD45 FITC 30-F11 Invitrogen FACS 1:400 AGM /YS lineage staining
CD45 PerCPCy5.5 30-F11 eBioscience FACS 1:400 Co-culture readout, AGM-HSC staining

CD45.1 APC- Efl 780 A20 eBioscience FACS 1:400 peripheral blood analysis
CD45.2 PerCp 5.5 104 eBioscience FACS 1:400 peripheral blood analysis
CD127 eF450 A7R34 eBioscience FACS 1:100 YS EMP/LMP staining
CD127 PE A7R34 Invitrogen FACS 1:100 YS EMP/LMP staining
CD48 APC, FACS sort LT-HSC HM48-1 eBioscience FACS 1:200 FL HSC staining
CD150 PE-Cy7 TC15-12F12.2 BioLegend FACS 1:400 FACS sort LT-HSC
CD150 PE TC15-12F12.2 BioLegend FACS 1:400 FL HSC staining
CD201 (EPCR,ProcR) APC eBio1560 eBioscience FACS 1:200 AGM-HSC
c-Kit APC 2B8 eBioscience FACS 1:400 AGM/YS EHT staining
c-KIT PE 2B8 eBioscience FACS 1:400 AGM-HSC / AGM staining
c-Kit APC eF780 2B8 eBioscience FACS 1:200 AGM/YS EHT, AGM-HSC and FL HSC staining
c-Kit SB436 2B8 eBioscience FACS 1:600 AGM/YS EHT staining
Ly-6G / Gr1 APC RB6-8C5 eBioscience FACS 1:400 Co-culture readout
Ly-6G / Gr1 FITC RB6-8C5 eBioscience FACS 1:400 YS lineage staining
Ly-6G / Gr1 PECy7 RB6-8C5 eBioscience FACS 1:200 Co-culture readout
LYVE1 eF660 ALY7 eBioscience FACS 1:10000 YS EHT staining
Ly-6G / Gr1 biotin RB6-8C5 eBioscience FACS 1:200 Fetal liver HSC, FACS sort LT-HSC
CD11b / Mac1 APC M1/70 eBioscience FACS 1:400 Co-culture readout
CD11b / Mac1 PE M1/70 eBioscience FACS 1:200 Co-culture readout
CD146 / MCAM Biotin ME-9FI BD FACS 1:200 YS EHT staining
CD146 / MCAM BV786 ME-9FI BD FACS 1:200 YS EHT staining
NK1.1 PerCpCy5.5 PK136 eBioscience FACS 1:200 Co-culture readout
Ly-6A/E (Sca-1)-FITC E13-161.7 BD FACS 1:200 AGM-HSC, Fetal liver HSC
Streptavidin BV421   Biolegend FACS 1:400 AGM/YS EHT staining
Streptavidin PerCP5.5 eBioscience FACS 1:200 Fetal liver HSC
SYTOX Green   Invitrogen FACS 1:1000 Viability dye
TER119 Biotin TER-119 Biolegend FACS 1:400 AGM /YS lineage staining, FACS sort LT-HSC
TER119 BV650 TER-119 Biolegend FACS 1:100 Co-culture readout
TER119 FITC TER-119 Biolegend FACS 1:400 AGM /YS lineage staining
TER119 PE TER-119 Biolegend FACS 1:200 Co-culture readout
Thy1.2 (CD90.2) SB600 53-2.1 Invitrogen FACS 1:200 Co-culture readout
Lyve1 AF2125 R&D Systems Whole mount IF 1:200 detection of HE-EMP
Runx1+Runx2+Runx3 EPR3099 Abcam Whole mount IF 1:400 detection of HE
GFP A10262 Invitrogen Whole mount IF 1:500 detection of Vwf:GFP reporter
anti goat Alexa Fluor Plus 647 A32849 Invitrogen Whole mount IF 1:500 detection of LYVE1
anti rabbit Alexa Fluor 555 A32794 Invitrogen Whole mount IF 1:500 detection of RUNX
anti chicken Alexa Fluor 488 A78948 Invitrogen Whole mount IF 1:500 detection of GFP

Validation

All antibodies used have been validated by the manufacturers for their respective applications.

Eukaryotic cell lines

Policy information about cell lines and Sex and Gender in Research

Cell line source(s)

OP9 cells (mouse bone marrow stromal cell line; ATCC® CRL-2749™) were obtained from the American Type Culture Collection (ATCC, Manassas, VA, USA).

Authentication

not done

Mycoplasma contamination

not tested

Commonly misidentified lines (See ICLAC register)

NA

Animals and other research organisms

Policy information about studies involving animals; ARRIVE guidelines recommended for reporting animal research, and Sex and Gender in Research

Laboratory animals

mouse embryos E9 - E16, were genereated from crosses between reporter male (C57BL/6JOlaHsd) mice and wt female Hsd:ICR (CD-1®) mice. For transplantations NSGS (NOD.Cg-Prkdcscid Il2rgtm1Wjl Tg(CMV-IL3,CSF2,KITLG)1Eav/MloySzJ) mice (CD45.1) aged 8-12 weeks were used.

Wild animals

No

Reporting on sex

Embryos were not sexed. adult transplant recipient were female

Field-collected samples

na

Ethics oversight

Mouse work was performed in accordance with the United Kingdom Animal Scientific Procedures Act (ASPA) 1986. Animal experiments performed at the Cancer Research United Kingdom Manchester Institute (CRUK-MI) were approved by the Animal

Welfare and Ethics Review Body (AWERB) of the CRUK-MI. Experiments performed at the University of Oxford were approved by the Oxford Clinical Medicine Ethical Review Committee.

Note that full information on the approval of the study protocol must also be provided in the manuscript.

Plants

Seed stocks	na
Novel plant genotypes	na
Authentication	na

Flow Cytometry

Plots

Confirm that:

☒ The axis labels state the marker and fluorochrome used (e.g. CD4-FITC).

☒ The axis scales are clearly visible. Include numbers along axes only for bottom left plot of group (a 'group' is an analysis of identical markers).

☒ All plots are contour plots with outliers or pseudocolor plots.

☒ A numerical value for number of cells or percentage (with statistics) is provided.

Methodology

Sample preparation	For the single cell RNA-seq and in vitro functional assays, dissected yolk-sacs were digested in a mix of Collagenase IV (2 mg/ml, Worthington) and DNase I (200 U/ml, Calbiochem) at 37 °C for 15 min. The dissociated cells were pelleted (300 g for 5 min at 4°C) and resuspended in phosphate buffered saline containing 10% fetal bovine serum (10% FBS in PBS) and further processed for FACS-analyses/sorting. E16.5 Fetal livers, dissected livers were crushed with the end of a 1ml syringe through a 40um cell strainer into IMDM+10% FBS. Dissected AGM tissues at E10.5 were finely chopped and the obtained fragments were digested in a mix of Collagenases IV (2 mg/ml, Worthington) and DNase I (200 U/ml, Calbiochem) at 37 °C with gentle agitation for 15 min. The dissociated cells were centrifuged at 300 g for 6 min and resuspended in PBS supplemented with 10% FBS for subsequent processing.<br>All flow cytometry analyses was performed on a BD LSRFortessa™ X-20 Cell Analyzer (BD Biosciences) or Novocyte Quanteon (Agilent). All cell sorting was performed on a BD FACSAria™ III Cell Sorter (BD Biosciences). Antibodies used for FACS are listed in table 5. For scRNA-seq cells were directly sorted into lysis buffer and snap-frozen before further processing. FlowJo software (BD Biosciences) was used to analyze all FACS data.
Instrument	BD LSRFortessa™ X-20 Cell Analyzer (BD Biosciences). BD FACSAria™ III Cell Sorter (BD Biosciences). Novocyte Quanteon (Agilent).
Software	BD FACSDiva™ Software, FlowJo v10
Cell population abundance	see manuscript
Gating strategy	see manuscript. extended figure 1

☒ Tick this box to confirm that a figure exemplifying the gating strategy is provided in the Supplementary Information.
